# Effect of digital health interventions on glycaemic control among type 2 diabetes individuals in low and middle-income countries: a systematic review and meta-analysis

**DOI:** 10.1186/s12889-026-27908-x

**Published:** 2026-06-08

**Authors:** Sherize Merlin Dsouza, Sahana Shetty, Renuka Suvarna, Prachi Pundir, Priyobrat Rajkhowa, Melissa Glenda Lewis, Helmut Brand

**Affiliations:** 1 Department of Global Public Health Policy and Governance, Prasanna School of Public Health, Manipal Academy of Higher Education, Manipal, Karnataka India; 2 Department of Endocrinology, Kasturba Medical College, Manipal Academy of Higher Education, Manipal, Karnataka India; 3 Collaboration for Evidence Synthesis (C4ES), Navelim, Goa India; 4 The George Institute for Global Health, New Delhi, India; 5 Division of Data Management and Biostatistics, Ministry of Health and Family Welfare, ICMR-National Institute for Research in Bacterial Infections (ICMR-NIRBI), Kolkata, India; 6 Department of International Health, Care and Public Health Research Institute – CAPHRI, Faculty of Health, Medicine and Life Sciences, Maastricht University, Maastricht, The Netherlands; 7 Prasanna School of Public Health, Manipal Academy of Higher Education, Manipal, Karnataka India

**Keywords:** mHealth, Digital Health Interventions, Type 2 diabetes mellitus, Diabetes self-management, Diabetes self-management application

## Abstract

**Background:**

Type 2 diabetes mellitus (T2DM) poses a significant public health challenge, particularly in Low- and middle-income countries (LMICs), where access to continuous Diabetes self-management (DSM) is limited. Digital Health Interventions (DHI) have emerged as promising tools to enhance DSM by facilitating self-monitoring and promoting health-promoting behaviours. This systematic review aimed to evaluate the effectiveness of DHIs in improving glycaemic control and their impact on health-promoting behaviours among individuals with T2DM in LMICs.

**Methods:**

A systematic review and meta-analysis of RCTs published between 2016 and 2025 was conducted to evaluate the effectiveness of DHI in managing blood glucose levels among individuals with T2DM aged ≥ 18 years. Databases such as PubMed, EMBASE, CINAHL, Scopus, Web of Science, and Cochrane Central Register of Controlled Trials (CENTRAL) were used for the search. Continuous outcomes were analysed using a random-effects model and reported as mean differences (MDs) with 95% confidence intervals (CIs). Risk of bias (ROB) was assessed using the Cochrane RoB 2 tool, and all analyses were performed in Review Manager (RevMan) version 5.4.

**Results:**

DHI demonstrated a significant improvement in glycaemic control across 15 of the total 23 included studies, with a reduction in HbA1c levels [MD: -0.58 (95% CI: -0.83, -0.33), *P* < 0.0001, I^2^ =98%]. Subgroup analysis conducted on the duration of intervention on HbA1c showed significant results at 3months [MD: -0.54 (95% CI: -0.85, -0.23), *P* = 0.0006, I^2^ = 88%] and ≥ 12 months [MD: -0.45 (95% CI: -0.63, -0.27), *P* < 0.00001, I^2^ = 93%]. Significant changes were observed in the subgroup analysis of the type of interventions and secondary outcomes assessed, such as Fasting Blood Glucose (FBG), Low-density lipoproteins (LDL), and Triglyceride (TG).

**Conclusion:**

The findings of this systematic review indicate that DHI can significantly improve glycaemic control among individuals with T2DM. mHealth applications and SMS reminders, as well as video-based interventions, can significantly improve health outcomes for individuals with T2DM. These digital tools support better self-management by promoting medication adherence, encouraging healthy lifestyle behaviours, and providing accessible education and motivation. SMS reminders help patients stay consistent with treatment plans and monitoring routines, while video interventions offer engaging and easy-to-understand guidance on diabetes care. The technologies also enhance patient engagement by improving glycaemic control and contribute to overall DSM.

**Supplementary Information:**

The online version contains supplementary material available at 10.1186/s12889-026-27908-x.

## Introduction

Diabetes Mellitus comprises a group of metabolic disorders characterised by persistent hyperglycaemia arising from impairments in insulin secretion, defects in insulin action, or a combination of both [1]. Type 2 Diabetes Mellitus (T2DM) represents the most prevalent of all types of Diabetes and poses a significant global public health challenge.

According to the International Diabetes Federation Diabetes Atlas, approximately 11.1% of the global adult population is living with type 2 diabetes, with projections indicating an increase to 853 million cases by 2050. Notably, nearly 40% of individuals remain unaware of their condition, with the burden of undiagnosed diabetes disproportionately higher in low- and middle-income countries (LMICs) [2]. 

Early identification of T2DM enables the initiation of evidence-based interventions, and research over the past three decades has demonstrated that progression from intermediate hyperglycaemia to T2DM can be delayed or prevented. However, the ability to identify individuals with undiagnosed T2DM and intermediate hyperglycaemia, and to implement effective diabetes prevention programs, varies widely across countries and healthcare systems, particularly in LMICs [3]. 

The prevalence of undiagnosed diabetes is highest in low-income countries (58.7%), followed by middle-income (45.5%) and high-income countries (28.9%). Preventive care coverage remains suboptimal in LMICs, where less than half of the population receives counselling on physical activity, diet, or weight management, and only a minority undergo routine blood glucose screening. These disparities are further compounded by systemic challenges within healthcare systems [2, 4] Hence, the primary focus of the geographical context covered in this study is limited to LMICs.

The World Health Organisation (WHO) also estimates that 38 million people die every year of chronic diseases, 85% of which are from LMICs. This can lead to delayed diagnosis, a rise in complications, and a higher burden on healthcare systems [5]. Multimorbidity, particularly among older adults, significantly elevates mortality risk and complicates disease management. A critical shortage of healthcare professionals, estimated at 4.3 million across 57 developing countries, further limits access to care, especially in rural and underserved regions [6, 7]. The response to these challenges can be Digital Health Interventions (DHIs), which have emerged as a promising approach to improving diabetes care.

The unprecedented spread of mobile technologies and advancements in their innovative application to address health priorities have evolved into a new field of eHealth, known as mHealth. The Global Observatory for eHealth (GOe) defined mHealth or mobile health as “medical and public health practice supported by mobile devices, such as mobile phones, patient monitoring devices, Personal Digital Assistants (PDAs), and other wireless devices” [8]. mHealth applications include the use of digital devices to gather clinical and community health- related data, provide healthcare information to patients, researchers, and practitioners, monitor patient vital signs in real time, and provide direct patient care [8]. 

The increasing prevalence of T2DM is closely associated with modifiable risk factors, including obesity, unhealthy diet, physical inactivity, and tobacco use, which are influenced by broader socioeconomic and environmental determinants [9]. Effective Diabetes self-management (DSM) requires an integrated approach combining pharmacological treatment with DSM strategies like lifestyle modifications, such as maintaining a healthy diet, engaging in regular physical activity, achieving weight control, and smoking cessation, which are essential for optimal glycaemic control. Behavioural factors, including medication adherence, nutrition, and physical activity, play a crucial role in influencing glycaemic outcomes, particularly glycated haemoglobin (HbA1c) levels [10]. 

In recent years, there has been an increasing number of DHIs meant to help T2D patients manage their condition, but only a few have been thoroughly evaluated among the general population globally [11]. A deeper knowledge of the influence of DHIs in controlling blood glucose levels and managing diabetes is crucial for DSM, especially in LMICs.

Digital health solutions have gained increasing attention as tools to support self-management and improve glycaemic outcomes in recent times. Although numerous applications have been developed for diabetes care, only a limited number have undergone rigorous evaluation, particularly in LMIC settings. Therefore, a comprehensive understanding of the effectiveness of these DHIs in improving glycaemic control is essential.

Given the rising global burden of T2DM and the significant gaps in early detection, prevention, and management, especially in LMICs, there is a need for innovative, scalable, and cost-effective solutions. Digital health interventions offer considerable potential to address these challenges by improving access to care, enhancing DSM, and supporting healthcare systems with limited resources.

Therefore, this study aims to evaluate the effectiveness of DHIs in improving HbA1c among individuals with T2DM. The findings are expected to contribute to evidence-based decision-making and inform the development of contextually appropriate, technology-driven strategies for diabetes self-management.

Therefore, this systematic review tried to address the problems identified from the protocol stage [12], such as “Are digital health interventions effective in managing blood glucose levels among individuals with type 2 diabetes mellitus in LMICs?” and, if yes, “What is the impact of using DHI in managing T2DM concerning health-promoting behaviour among the LMICs” [12].

## Methods

The Preferred Reporting Items for Systematic Review and Meta-analysis (PRISMA) 2020 guidelines [13] are used for reporting the systematic review. The systematic review protocol was registered in PROSPERO (CRD42021245517).

### Study selection

Randomised Controlled Trials involving adults aged ≥ 18 years who used a smartphone or personal computer, and were diagnosed with T2DM, according to WHO 2020 criteria [14] [HbA1c ≥ 6.5% or fasting plasma glucose (FPG) ≥ 7.0 mmol/L (126 mg/dL)] were included. The intervention group comprised individuals receiving a DHI, while the comparator group received standard diabetes care without any digital component. Standard care generally is comprised of pharmacological management and basic lifestyle advice, without the use of digital or app-based interventions in the context of LMICs. Eligible studies were conducted in LMICs as per World Bank 2024 classification criteria, published in English language (due to the lack of resources for translation and the need to ensure accurate interpretation of study findings, we limited the language to English as it is also a dominant language of publication for many international scientific journals, ensuring relevant evidence being captured), and assessed the effects of any kind of DHIs, including apps, text messages and reminders, email, video clips, graphics, and web services, for diabetes self-management. Countries classified as LMICs in this meta-analysis were defined according to the World Bank 2024 classification criteria. Studies that did not include DHIs, or enrolled participants who were not diagnosed with diabetes were excluded.

### Search strategy

PubMed, EMBASE, CINAHL, Scopus, Web of Science, and Cochrane Central Register of Controlled Trials (CENTRAL) database were used for study screening. The search included publications published between January 2016 and May 2025 in the English language. We have also searched the references of included studies as a part of the search strategy. The keywords used for the search strategy are shown in the supplementary file (Refer: Supplementary file 1). Each title was individually screened by two authors following the pre-set inclusion criteria before being included in the systematic review. Two authors independently assessed the included articles’ titles and abstracts from the initial screening phase, and the authors then reviewed the included studies for full text. If the authors’ conclusions did not agree at either of the screening stages, the third author made the final decision. Reasons for exclusion are given at the full-text screening stage, and the PRISMA flow diagram is used to depict the screening process.

#### Intervention

Digital Health Interventions (DHI), including mHealth apps and wireless technologies, are used to support efforts aimed at attaining our objectives. Digital health describes “the general use of information and communications technologies (ICT) for health and is inclusive of both mHealth and eHealth” [15].^.^ From the context of this study, the term mHealth refers to the mobile applications used in the self-management of T2DM. The interventions in our study included mHealth apps and other digital solutions like text messages, email, video clips, graphics, and web services.

### Data extraction and outcome measures

Two authors generated a standardised data extraction form in Microsoft Excel and pilot tested it on two included studies by two independent reviewers. Discrepancies were discussed, and the fields for extraction were refined by two individual reviewers who interpreted the fields consistently by reviewing and coming to a consensus, which was later approved by the third reviewer. The form captured all the needed data. The form included information on the studies’ characteristics, geographical region, population, intervention, and clinical outcomes. The primary outcome assessed is the HbA1c. The subgroup analysis has been conducted for the duration of the given DHI at 3, 6, and ≥ 12 months, and for the type of DH interventions, including mHealth, voice, and SMS. Secondary outcomes assessed were Fasting Blood Glucose (FBG), Body Mass Index (BMI), Total Cholesterol (TC) Levels, High-Density Lipoprotein (HDL), Low-Density Lipoprotein (LDL), Triglyceride Levels (TG), Blood Glucose Monitoring (BGM), Physical Activity, and Medication adherence.

While the secondary outcomes were not explicitly specified as secondary study outcomes to be analysed in the protocol stage, we have added a few secondary outcomes as they emerged as clinically relevant and as they were consistently reported across several included studies during the data extraction.

### Assessment of risk of bias

The Cochrane Risk of Bias (RoB-2) tool was used to evaluate the study quality of the randomised controlled trials [16]. Two authors independently assessed the risk of bias in the included studies, and the conflicts were resolved by consensus. The rating for RoB2 was ‘low risk of bias’, some concerns, and ‘high risk of bias’.

### Data synthesis

A comprehensive narrative of the outcomes of intervention, medication adherence and self-efficacy is presented. For homogenous data, a meta-analysis of the continuous outcomes was conducted. In instances where the mean and standard deviation of the change from baseline to endpoint were not reported in the original articles, the following equations were employed to calculate these values,

$$\:\:Change\:in\:Mean={Mean}_{T2}-{Mean}_{T1}\:$$

$Change\;in\;SD=\sqrt{\left(SD_{T1}\right)^2{+\left(\right)}\left(SD_{T2}\right)^2-{2rSD_{T1}SD_{T2}}}$ where T_1_ and T2 are the baseline and endpoint values, and r is the correlation coefficient (r) taken as 0.4 [17]. A random effects model (inverse variance method) was used, and the I² statistic was used to measure heterogeneity. Publication bias was evaluated using funnel plots, and further Egger’s regression test with ≥ 3 studies was performed to detect small-study effects. The meta-analysis was performed using RevMan 5.4 software.

## Results

**Fig. 1:**
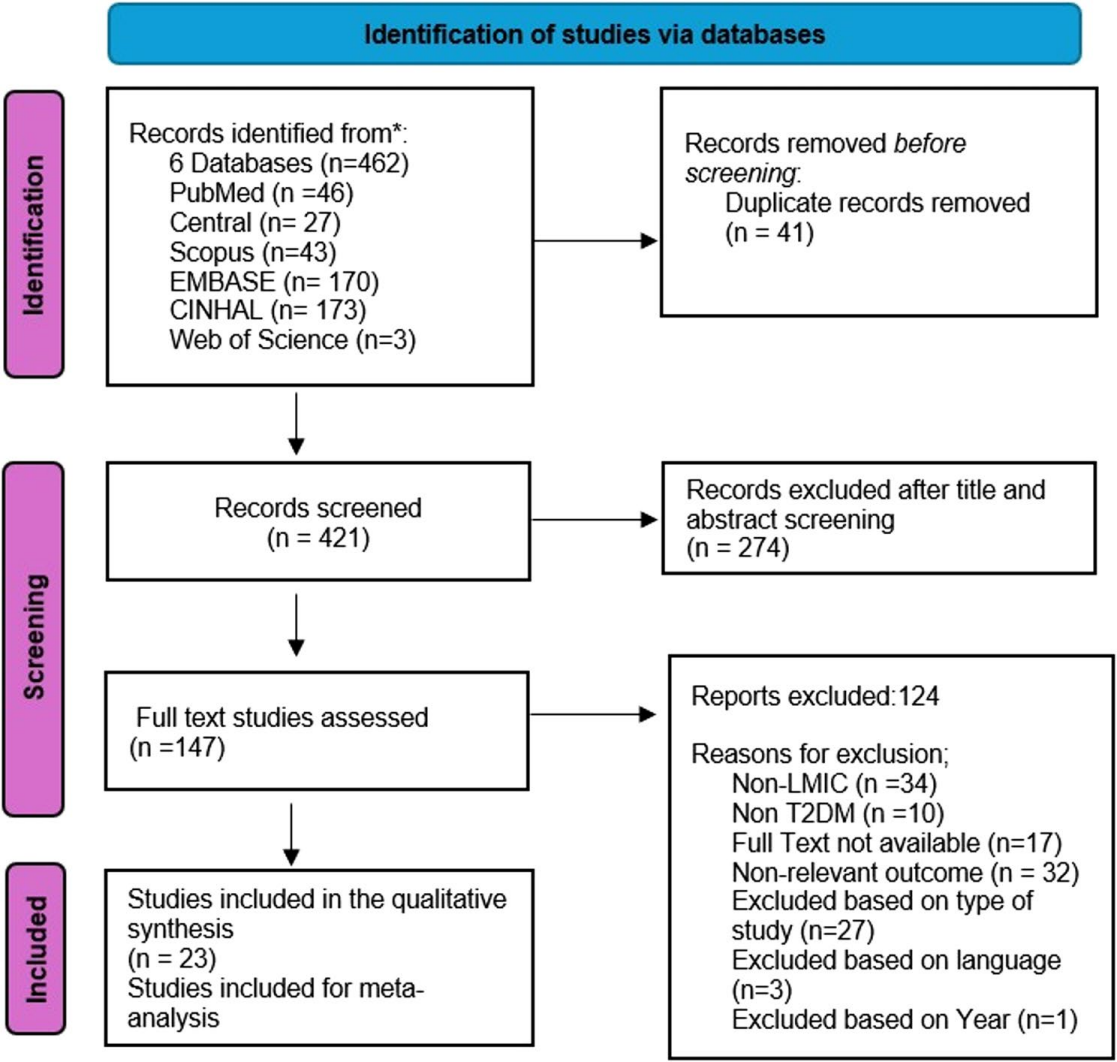
PRISMA flow diagram depicting the study selection and inclusion

Initial searches identified 462 articles from six databases listed in the methods. Of the 147 articles included in full-text screening, 124 were excluded for failing to meet the inclusion criteria. Finally, 23 articles were eligible for the review, and a meta-analysis was performed on the pooled data. (Refer: Fig. 1).

### Characteristics of included studies

The review included 23 studies [18–40]: 22 randomised controlled trials (RCTs) and one non-randomised controlled trial (NRCT), involving 27,807 participants from LMICs (Refer: Supplementary file 2). All studies implemented some form of educational DHI lasting between 3 and 24 months.

Nine studies used DHIs, including educational videos delivered through the app, while 6 studies used other non mHealth Digital interventions (such as SMS and voice) that relied on text messages, reminders, and educational materials delivered via videos.

For the meta-analysis, data were extracted for the intervention and control groups as reported in the included studies. In studies with more than two groups [40], the four study arms were collapsed into two groups for analysis: a combined intervention group (with app and with or without DSME) and a combined control group (DSME without mHealth app and usual care). Another study [32], ^,^ combined the four groups to form an intervention group with a Mobile Phone application and with or without Self-Management Blood Glucose (SMBG) education. While the control group consisted of either no SMBG or the presence of SMBG alone, without the mHealth app. The groups were categorised according to the type of intervention and control conditions as mentioned in the inclusion criteria of this study. Where appropriate, relevant groups were combined to derive pooled mean change values for the intervention and control arms.

### Meta-analysis

#### Glycated Haemoglobin (HbA1c)

**Fig. 2:**
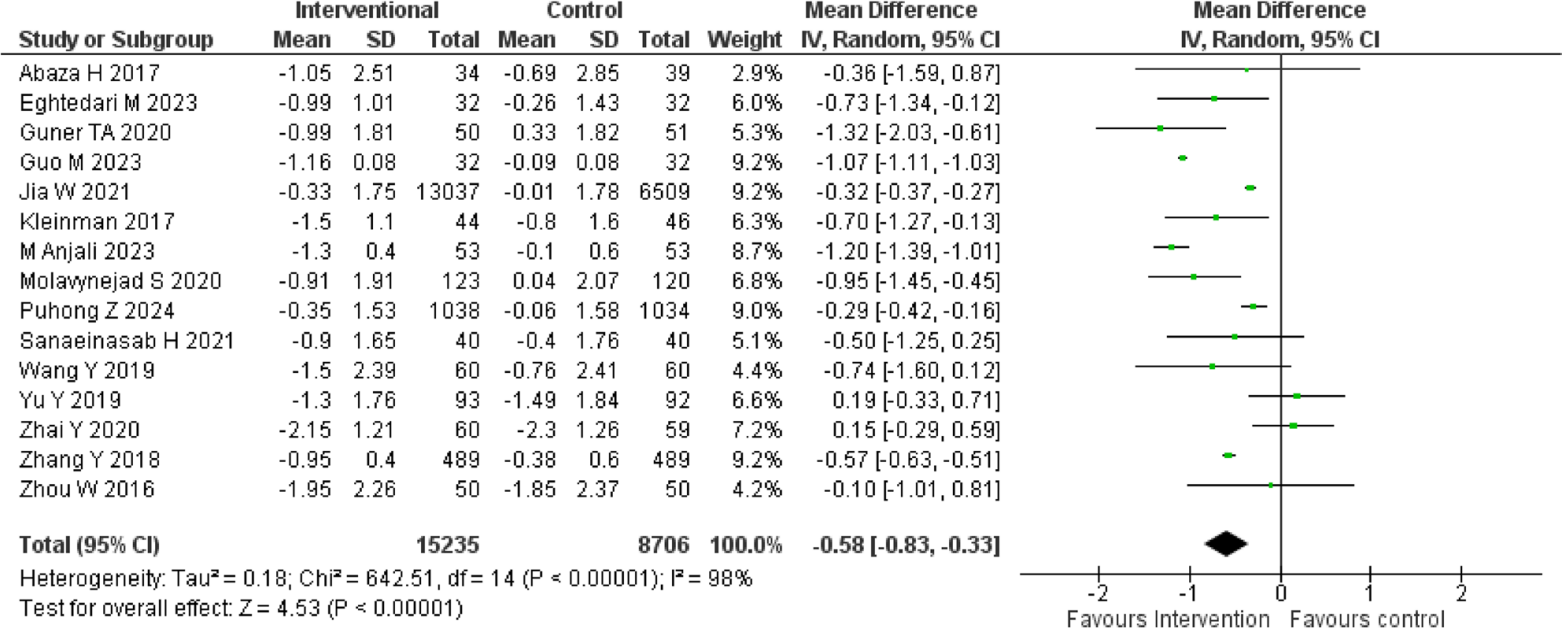
Forest plot showing the change in HbA1c levels in the Digital Health Intervention group compared to the control group

Fifteen studies were included in the meta-analysis of HbA1C with a total of 27,807 participants. The DHI group consisted of 15,235 participants, while the control group comprised 8,706 participants. The intervention significantly reduced HbA1C compared with control [MD: -0.58% (95% CI: -0.83, -0.33), *P* < 0.00001, I^2^= 98%] with substantial heterogeneity (I^2^) among studies (Fig. 2).

### Subgroup analysis of HbA1c

**Fig. 3:**
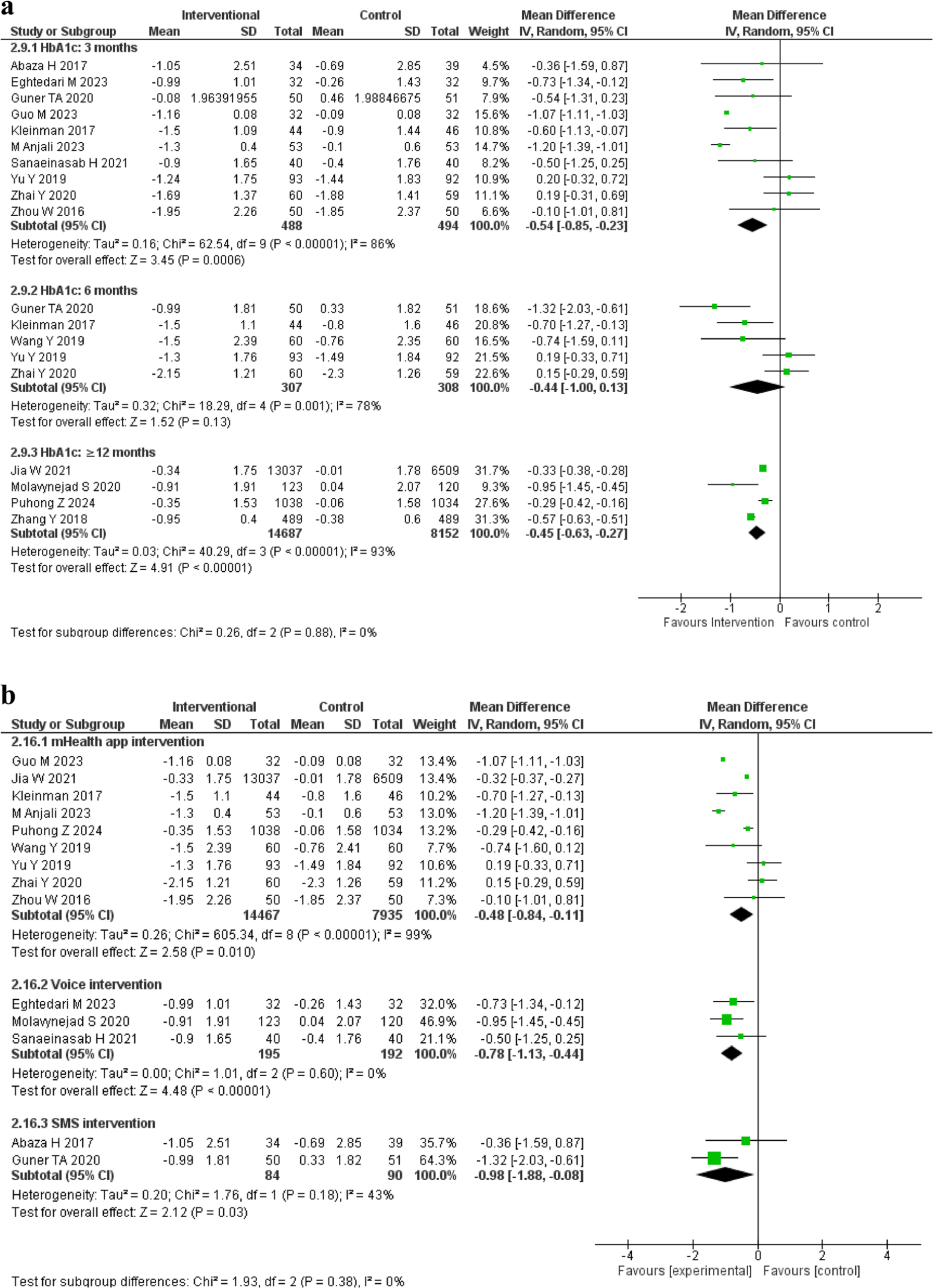
**a** Forest plot illustrating the effect of DHI on HbA1c levels compared to the control group across different follow-up durations. **b **Forest plot showing the mHealth app, Voice and SMS interventions in the Intervention group and the control group

Duration of Intervention: At 3 months, ten eligible studies (488 intervention; 494 control) showed a significant reduction in HbA1c [MD -0.54% (95% CI -0.85, -0.23), *P* = 0.0006; I² = 86%]. At 6 months, five studies (307 intervention, 308 control) demonstrated a non-significant result [MD -0.44% (95% CI -1.00, -0.13), *P* = 0.13; I² = 78%]. At 12 months and greater duration of intervention, four studies (14687 intervention; 8152 control) showed a highly significant reduction [MD -0.45% (95% CI -0.63, -0.27), *P* < 0.00001; I² = 93%]. (Refer: Fig. 3a).

Type of interventions: Of the 15 studies included for meta-analysis on HbA1c as the primary study outcome, nine studies used an mHealth app as the intervention, with 14,467 intervention participants and 7935 control participants, showing significant changes in the intervention group as compared to the control group, [MD -0.48% (95% CI -0.84, -0.11), *P* = 0.01; I² = 99%]. Similarly, six studies (768; intervention, 771; control) not using an mHealth app reported to have [MD -0.69% (95% CI -1.91, -0.47), *P* < 0.00001; I² =26%], showing highly significant differences in the intervention group (Refer: Fig. 3b).

While the effect of the subtype type of non-mHealth interventions was possible to be pooled for two of the intervention subtypes, namely, SMS and video interventions. The SMS intervention did not show significant improvement in two of the studies included for the meta-analysis, with [MD -0.98 (95% CI -1.88, -0.08), *P* = 0.03; I²= 43%], while the video-based interventions have shown to be statistically significant, favouring the intervention [MD -0.78 (95% CI -1.13, -0.44) *P* < 0.00001; I²= 0%]. (Refer: Fig. 3b)

#### Secondary outcomes

##### Fasting blood sugar

Twelve trials (14,704 intervention; 8,170 control) were included. The intervention significantly reduced FBG compared to the control group [MD -0.50 mmol/L (95% CI: -0.84, -0.17), *P* = 0.003], with I² of 78% (Refer: supplementary file 3, Fig. 4).

##### Body mass index

Nine trials were included in the meta-analysis of BMI, with 1266 participants in the DHI group and 1240 participants in the control group. The forest plot did not show significant changes in BMI between the groups, with moderate heterogeneity [MD -0.09 kg/m^2^ (95% CI -0.37, 0.20), *P* = 0.56] detected among studies (I² =40%) (Refer: supplementary file 3, Fig. 5).

##### Lipid profiles

A meta-analysis of four trials (702 intervention; 700 control) showed no effect on HDL [MD 0.97 mg/dl (95% CI: -4.07, 6.00), *P* = 0.71], with moderate heterogeneity I^2^ =79% across the studies (Refer: supplementary file 3, Fig. 6).

LDL levels assessed in seven trials (14,827 intervention; 8293 control) showed a highly significant difference between groups [MD -2.10 mg/dl (95% CI -2.90, -1.30), *P* < 0.00001, I^2^ = 99%] (Refer: supplementary file 3, Fig. 7).

Triglyceride levels assessed in seven trials (213 intervention; 211 control) showed a significant difference between groups [MD -18.15 mg/dl (95% CI -34.18, -2.11), *P* = 0.03, I^2^ = 0%] (Refer: supplementary file 3, Fig. 8).

Total Cholesterol levels assessed in three trials (651 intervention; 646 control) showed no significant difference between groups [MD -1.03 mg/dl (95% CI -11.58, 9.53), *P* = 0.85, I^2^ = 68%] (Refer: supplementary file 3, Fig. 9).

##### BGM

Four trials assessing BGM were included, involving 231 participants in the intervention group and 236 in the control group. No significant change was observed in BGM levels in the DHI compared to the control group [MD -0.25 (95% CI -1.12, 0.62), *P* = 0.58, I² = 82%], accompanied by substantial heterogeneity among studies (Refer: supplementary file 3, Fig. 10). BGM was assessed primarily through self-reported questionnaires.

##### Physical activity

Five trials were included in the study, with 218 participants in the intervention group and 219 participants in the control group. The forest plot did not show significant changes in the physical activity levels between the groups [MD 0.33 (95% CI -0.81, 1.46), *P* = 0.57, I ^2^ = 100%] with substantially high levels of heterogeneity among the studies (Refer: supplementary file 3, Fig. 11). In the studies included, Physical activity was assessed primarily through self-reported questionnaires, except for 2 studies, where physical activity was assessed by accelerometers and a cloud data management system).

### Medication adherence

Four trials were included in the study, with 170 participants in the intervention group and 175 participants in the control group. The forest plot did not show significant changes in the medication adherence levels between the groups [MD -0.38(95% CI -1.18, 0.41), *P* = 0.34, I ^2^ = 83%] with substantial levels of heterogeneity among the studies (Refer: supplementary file 3, Fig. 12). In the studies provided, medication adherence was assessed primarily through self-reported questionnaires rather than objective measurements.

### Heterogeneity and publication bias

The visualization of funnel plot demonstrated noticeable asymmetry for most of the outcomes (Refer: Supplementary file 3, funnel plots: S1-S10).

The Cochrane Q test and I2 statistics revealed significant between-study heterogeneity for a number of outcomes (Refer: Supplementary file 4, Table 1). For the majority of outcomes, including HbA1c, FPG, total cholesterol, HDL, LDL, triglycerides, BGM, physical activity and medication adherence, Egger’s test revealed no evidence of publication bias. However, the Egger’s test was inconsistent in BMI (*p* = 0.036), indicating that the observed asymmetry may not be solely attributable to publication bias. The variation might possibly have been caused by other factors like methodological differences, small-study effects, and between-study heterogeneity.

### Sensitivity analyses

The pooled estimates were typically robust across outcomes, according to sensitivity analyses (Refer: Supplementary file 4, Table 1). After sensitivity analysis, the effect sizes for HbA1c and BMI remained unchanged, indicating that no single study had a disproportionate impact on the outcomes. Similarly, lipid outcomes (Total Cholesterol, HDL, and Triglycerides) showed negligible changes in effect estimates, confirming the stability of these findings.

For FPG, although the direction of effect was preserved, the confidence interval widened in sensitivity analysis; the exclusion of individual studies [20, 36, 39] contributed to this variation. A similar pattern was observed for LDL, where exclusion of two studies [34, 22] indicated reduced robustness of the pooled estimate. For BGM, physical activity and medication adherence, the result remained non-significant with negligible changes in effect size, indicating consistent findings.

### Risk of bias in included studies

**Fig. 4:**
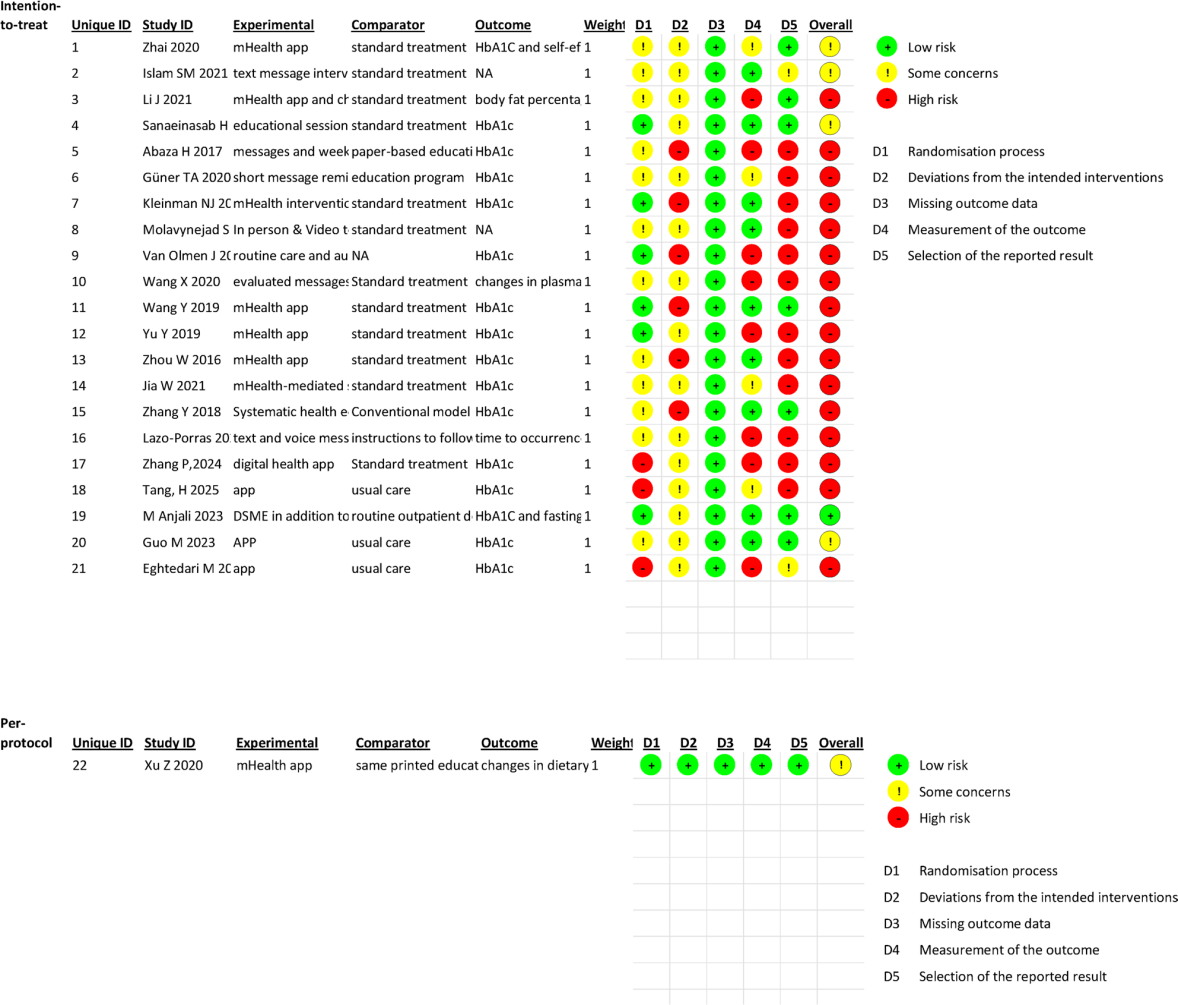
Risk of bias summary on judgments of each RoB2 item for each included study

Twenty-three included studies in this systematic review were inclusive of 22 RCTs and one NRCT. Most of the RCTs were assessed as having some concerns or High risk in the randomisation process (Refer: Fig. 4). One of the studies [39], scored a low risk of bias in the overall rating, with 16 studies having an overall high risk of bias [18, 20, 22–25, 27–30, 32, 34, 35, 37, 38, 40] in the intention to treat analysis, and four studies [21, 26, 33, 36]^,^ included in intention to treat analysis and one study with overall risk of bias rating low risk. One of the included studies [31] scored some concerns in the overall risk of bias rating in the per-protocol analysis. This overall pattern suggests that the internal validity of the included evidence is limited.

At the domain level 1, the randomisation process is frequently rated as having some concerns, primarily due to insufficient reporting of allocation concealment, randomisation process and insufficient baseline comparability among the participants. At Domain 2 and 3, Deviations from intended interventions and missing outcome data were less problematic overall, with most studies rating some concerns in Domain 2 and all studies assessed as having low risk of bias in Domain 3. Domains 4 and 5, measurement of the outcome and selection of the reported result, emerged as the most common sources of bias, with many studies rated as high risk in domains 4 and 5. These issues were largely attributable to a lack of blinding, reliance on self-reported measures, and potential selective outcome reporting. Collectively, these limitations may have introduced bias in effect size estimates and reduced confidence levels in the overall evidence base.

### Certainty of evidence

The table (Refer: Supplementary file 4, Table 2) illustrates the certainty of evidence through the GRADE approach across outcomes, which ranged from moderate to very low. Despite precise and consistent effect estimates, HbA1c and FBG were both classified as low-certainty evidence for glycaemic outcomes, mainly because of concerns about bias and indirectness. Because of the significant possibility of bias, extreme imprecision, and suspected publication bias, BMI was rated as having very low certainty.

Total cholesterol and HDL were classified as having very poor certainty for lipid outcomes, mostly because of severe imprecision and indirectness. On the other hand, LDL, triglycerides and BGM demonstrated low-certainty evidence, with downgrading mostly caused by high risk of bias and inconsistency. Due to the possibility of high risk of bias, indirectness, and extremely serious imprecision, physical activity and medication adherence were classified as having very low certainty, with large confidence ranges signifying significant uncertainty. Overall, the certainty of evidence across outcomes ranged from low to very low, downgraded mainly due to methodological limitations, inconsistency, and imprecision.

### Qualitative synthesis of studies not eligible for HbA1c meta-analysis

One NRCT [19] in China demonstrated that an education and mHealth management model significantly decreased HbA1C levels and increased the proportion of patients achieving their target HbA1c goal, i.e. <7%. Another RCT [38] in Iran found that a web-based Diabetes self-management program significantly reduced HbA1c values.

Multiple DHI found no significant difference in HbA1c outcomes between interventions and control groups. One RCT in Bangladesh and the other in the Democratic Republic of Congo, Cambodia, and the Philippines [21, 28] reported that text messaging failed to significantly improve HbA1c levels or increase the percentage of patients with controlled HbA1c. Trials using fitness apps with wearable monitors in China [25] and foot thermometry with mHealth reminders in Peru [23] also reported no significant between-group differences in HbA1c reduction. Similarly, an mHealth app (Dnurse app) study in China [40] showed a tendency to improve HbA1c compliance rates for T2DM patients; however, the overall main effect did not show statistical significance.

Two studies conducted in China [29, 31] highlighted practical challenges associated with measuring HbA1c. One study [29] acknowledged HbA1c as a gold standard for assessing glycaemic control but relied on fasting and post-prandial glucose instead. Another study [30] did not measure biochemical markers like HbA1c to keep the costs low.

Self-efficacy was assessed by two studies [20, 33], that employed standardized and validated instruments using the Diabetes Self-Efficacy Scale and the Diabetes Self-Care Scale, respectively, providing comparable measures that are seen to improve outcomes post DHI in these studies.

## Discussion

This Meta-analysis showed evidence for the effectiveness of the DHIs in improving glycaemic control and promoting healthier lifestyle behaviours among individuals with diabetes. Participants in the DHI group showed a reduction in HbA1c compared to the control groups. A significant reduction in FBG, TG and LDL levels was observed in the DHI as compared to the control group, whereas no significant change in BMI, BGM, physical activity, Total cholesterol and HDL levels was observed. Individuals with T2DM who used DHIs showed greater reductions in HbA1c and FBG compared to those receiving standard care, similar to a systematic review study conducted in India on mHealth technology-enabled interventions to improve management of diabetes [41]. Also, the subgroup analysis done on the duration of intervention of HbA1c also indicated similar results found from another systematic review conducted in India [42]. In the analysis, although the overall test for subgroup differences by study duration was not statistically significant at 6 months, we observed that HbA1c reduction reached statistical significance at 3month and ≥ 12-month time points. This apparent discrepancy may be due to differences in sample size and study characteristics within each subgroup rather than a true effect modification by duration. In particular, the 6-month subgroup showed comparable effect estimates, but with wider confidence intervals, which may have limited the ability to detect statistical significance. These findings highlight DHIs as a key component of DSM strategies and reinforce their role in achieving improved clinical outcomes among individuals with T2DM, and can support patients’ overall well-being [43]. Although the meta-analysis of BMI and other secondary outcomes showed no significant effect, variations in the type and duration of interventions across studies limit the ability to determine specific impacts of DHIs on other behavioural outcomes and weight management [44]. 

Numerous research studies have demonstrated that there is often an initial improvement in glycaemic control and adherence to self-management behaviours, but sustaining these improved health outcomes can be difficult [45, 46]. As observed in our study, the subgroup analysis conducted is evident, showing the HbA1c outcomes improving significantly at the greater duration of intervention, i.e., ≥ 12 months. Whereas a lack of long-term follow-up and a potential for decreased adherence over time were also consistent with findings from a research study [47]^,^ while in another study, inconsistent adherence was measured in a few of the included studies measuring medication adherence [48]. Hence, it’s important to measure the medication adherence for longer follow-up durations to understand the pattern of adherence on the clinical outcomes. Reasons for lack of adherence could be caused by several factors in the given scenario of DHIs, including the possibility of the organisation’s lack of continued support for the DHIs. Given that digital applications are provided as part of intervention studies or have a short duration, there is a significant likelihood that participants would discontinue using them because they may involve subscription fees.

Similarly, few studies have also highlighted the need for long-term use of interventions to understand the actual effectiveness of the given interventions, which is in line with the findings observed from this meta-analysis [49, 50]. Of the 23 included studies, four studies have examined the sustained use of DHI over extended intervention periods (≥ 12 months), where the sustainability of DHI for DSM shows varying levels of long-term engagement and effectiveness. A study from China [37] demonstrated sustained engagement with a diabetes management mobile application over 24 months, with nearly half of participants remaining active monthly users and higher engagement associated with better glycaemic outcomes. In another study [34], long-term participation was maintained through continuous digital communication using the WeChat platform alongside structured health education over two years, supporting sustained self-management and improvements in metabolic indicators. Similarly, another randomised trial [28] implemented SMS-based diabetes self-management support for two years; although the intervention was maintained throughout the period, its sustained clinical impact on glycaemic control was moderate. Lastly, another study [24] demonstrated high long-term adherence to daily self-monitoring supported by SMS reminders for over 18 months, although the sustained engagement did not significantly reduce diabetic foot complication incidence. Overall, these studies suggest that DHIs can maintain long-term user engagement and adherence, but the extent of sustained clinical benefit varies depending on the intervention design, level of interaction, and integration with broader DSM strategies.

Therefore, the DHIs may be helpful when tailored to the needs of the organisation and distributed to individuals with diabetes to enable them to successfully treat and manage diabetes. If not, participants can find it difficult to sustain their new behaviours when the digital intervention support is not ongoing, due to which, adhering to healthy lifestyle habits can be challenging due to personal, social, and environmental factors, especially as diabetes progresses [51]. Clinicians can support self-management through effective communication and a well-integrated healthcare system. While some developed countries have made progress, most LMICs need institutional support to develop and implement national eHealth strategies in their routine [52] by promoting evidence-based DHIs, with regional adaptations for diabetes management, that align with the global digital health strategy for a smarter disease management. Effective mHealth app development must prioritise user needs, ensuring design, functionality, and testing that are aligned with the target population [53]. Ignoring these needs can result in apps that are difficult to use, irrelevant, or threatening.

A key strength of this review is that it provides valuable insights for healthcare providers and policymakers in advocating the use of DHIs, as a reduction of -0.58% in HbA1c is considered clinically significant, as it reflects an improvement in average blood glucose levels over the follow-up period. Even the slightest reductions in HbA1c are associated with a decreased risk of microvascular complications in individuals with T2DM. Therefore, a decline of this magnitude is regarded as a meaningful clinical improvement and may support the continuation of the current DHI by including mHealth applications for long-term use in the management of diabetes. To focus on populations with the greatest need and limited resources, we focused our analysis on LMICs where the disease burden is higher, and healthcare expenditure is lower. This regional focus highlights the growing feasibility and effectiveness of DHI in resource-constrained settings. The current analysis provides updated evidence and incorporates a larger dataset reflecting recent technological advancements, including smartphone-based monitoring, teleconsultations, and app-based education. Notably, this meta-analysis adds to the existing literature by including studies that integrate behaviour-change strategies, demonstrating that digital tools can reinforce healthier lifestyle practices.

The limitations include heterogeneity across the studies with respect to data collection methods, participant demographics presented, and follow-up durations, which might have led to bias in results. The majority of studies evaluating DHIs demonstrated a high overall risk of bias, as indicated by the risk of bias assessment. As a result, the analysis obtained may have discrepancies due to small sample sizes, short follow-up durations, studies lacking blinding on allocation, and reliance on self-reported outcomes without the standard use of a measurement tool, which may compromise the validity and reliability of the study findings. Therefore, well-designed, adequately powered trials are needed to confirm their effectiveness. Additionally, all included studies were conducted in LMIC settings, which may limit the generalizability of the findings to other populations. Another limitation is that the participants with limited digital skills may experience barriers to access and reduced adherence, potentially attenuating the observed effects. This disparity could lead to an overestimation or underestimation of the true intervention effect. Additionally, digital literacy is often associated with factors such as age, education, and socioeconomic status, which may further confound outcomes.

The findings of this meta-analysis may have implications for the design and execution of digital health initiatives, especially in LMIC contexts. Features that could improve engagement and efficacy include tailored content, user-friendly interfaces, SMS reminders, voice interventions and integration with standard clinical treatment. Given the significant variation in the study designs, participants, and intervention features, these findings should be viewed as exploratory rather than final recommendations. Future well-designed research is required to precisely assess the impact of these factors on the efficacy of interventions. Future research studies should incorporate behavioural change outcomes such as BMI, BGM, total cholesterol, HDL, and medication adherence, which were not found to be statistically significant in our study due to some of the reasons cited above, to provide a complete picture of the DHI’s effectiveness in these categories as well. The follow-up of participants over extended periods can help establish the long-term impact of behavioural changes on clinical outcomes. Understanding participants’ experiences of using apps to maintain their health can provide valuable insights into behaviour change processes. Health promotion essential through DHI pathways, facilitating early diagnosis, enabling timely treatment of disease, and aiming to reduce the risk of future complications and mortality associated with T2DM.

## Conclusion

This systematic review highlights the significant potential of DHIs in improving HbA1c as an outcome. The findings demonstrate that DHIs can enhance diabetes care through integrated strategies such as dietary guidance, physical activity tracking, BMI management, pharmacotherapy adherence, and continuous glycaemic monitoring. Despite challenging outcomes, further high-quality and long-term research is essential to overcome the limitations identified in the existing evidence and to determine the most effective, sustainable, and patient-centred digital interventions. Strengthening patient engagement and adherence remains critical to maximising the long-term benefits of DHIs. Collaborative efforts among healthcare professionals, policymakers, technology developers, and community stakeholders are therefore necessary to successfully integrate these DHIs within primary healthcare settings, with future expansion toward tertiary care systems. Such coordinated implementation has the potential to improve clinical outcomes, promote self-management, and reduce the overall burden of T2DM on healthcare systems.

## Supplementary Information

Supplementary Material 1.

Supplementary Material 2.

Supplementary Material 3.

Supplementary Material 4

## Data Availability

All data supporting the findings of this study are available within the paper and its Supplementary Information.

## Abbreviations

ADA: American Diabetes Association
BMI: Body Mass Index
BGM: Blood Glucose Monitoring
DSM: Diabetes Self-Management
DHIs: Digital health interventions
FBG: Fasting Blood Glucose
GOe: Global Observatory for eHealth
HbA1c: Glycosylated haemoglobin
HDL: High-Density Lipoprotein
ICT: Information and Communication Technologies
IDF: International Diabetes Federation
LDL: Low-Density Lipoprotein
PDAs: Personal Digital Assistants
PRISMA: Preferred Reporting Items for Systematic Reviews and Meta-Analyses
RCTs: Randomised Controlled Trials
SMBG: Self-Management Blood Glucose
SMS: Short Message Service
NRCT: Non Randomised Controlled Trial
RoB 2: The Cochrane Risk of Bias Tool 2
TC: Total Cholesterol
TG: Triglyceride Levels
T2D: Type 2 diabetes
T2DM: Type 2 diabetes mellitus
WHO: World Health Organization
