# Supplementary material for: Effect of digital health interventions on glycaemic control among type 2 diabetes individuals in low and middle-income countries: a systematic review and meta-analysis
Source: BMC Public Health. 2026 Jun 8;26:2340. doi: 10.1186/s12889-026-27908-x (PMC13463556; doi:10.1186/s12889-026-27908-x)
Supplement: Supplementary file 1 [file 12889_2026_27908_MOESM1_ESM.docx]

| **Sl. No.** | **Study ID** | **Country** | **Sample size (type 2 diabetic Population)** | **Average Duration of diabetes in years** | **Mean Age** | **Gender** | **Intervention** | **Control group/ comparator** | **Duration of intervention** | **Outcomes assessed** | **Baseline clinical data of HbA1c** | **Weight /** Body mass index. (**BMI)** | **Average frequency of blood glucose measurement** | **FPG** | **HDL** | **LDL** |
| --- | --- | --- | --- | --- | --- | --- | --- | --- | --- | --- | --- | --- | --- | --- | --- | --- |
| 1 | Abaza H 2017^18^ | Egypt | **Intervention (I)** –34  **Control (C)-**39 | (Duration of Diabetes ≥1)  **I-** 29(85.29)  **C-** 32(82.05) | **I**- 51.24±8.66  **C-** 51.77±9.68 | **Intervention:** Females-  (F)-18(52.94)  Males (M)-Not Reported (NR)  **Control:**  F- 23(58.97)  M-NR | Diabetes educational SMS messages, in addition to reminder prompts | Received a booklet of diabetes care instructions at the beginning of the study, but did not receive SMS messages | 3months | HbA1c | I- 9.78±2.53  C- 9.53±2.78 | WEIGHT –  I - 85.9±13.8  C- 89.9±17.8 | I-2.38±0.95  C-2.28±1.15 | NR | NR | NR |
| 2 | Chai S^19^ | China | I-209  C- NR | I- 5(1,10)  C- NR | I-59.0±10.6  C-NR | NR | video conference on diabetes education. | NA | 4months | HbA1c Fasting plasma glucose (FPG) and postprandial plasma glucose (PPG) | I-8.6±1.7  C-NR | BMI  I-26.00±2.84  C-NR | NR | I- 9.7(8.4,12.1) | I-1.3(1.1,1.5) | I-3.0(2.4,3.6) |
| 3 | Guner TA, 2025 ^20^ | Turkey | I-50  C-51 | I-7.86 ± 4.37  C-8.03 ± 5.05 | I-55.74 ± 8.57  C- 57.69 ± 9.15 | **Intervention** F- 54%  M- 46%  **Control-**  F-51  M- 49 | Education modules given by a Family health centres (FHC) along with short text message reminders | Education given by FHC only | 6 Months | HbA1c, Fasting Blood Sugar (FBS), lipid profile and BMI | I-7.58 ± 1.94  C-7.17 ± 1.72 | I- 32.13 ± 4.66  C-32.34 ± 5.33 | NR | I-177.84 ± 77.69  C-156.45 ± 55.98 | I-43.96 ± 11.78  C- 45.08 ± 10.83 | I-120.74 ± 40.61  C-121.51 ± 35.92 |
| 4 | Islam SM 2021^21^ | Bangladesh | I- 94  C-106 | NR | I-NR  C-NR | Intervention: F- NR  M-NR  Control:  F- NR  M-NR  (TOTAL)  F- 54.2%  M-45.8% | text message intervention | Usual hospital care | 6 months | The text messaging program did not significantly improve dietary behaviour in patients with T2DM. | NR | NR | NR | NR | NR | NR |
| 5 | Jia W 2021^22^ | China | **I-**13,037  **C-**6,509 | I-6.0(3.0,10.0)  C-6.0(3.0,11.0) | I-60.4(8.4)  C-60.8(8.4) | Intervention: F-NR  M-41.8%  Control:  F- NR  M- 40.1% | mHealth app-mediated service package using a provider-facing smartphone application Graded ROADMAP and a website. | Usual hospital care | 12 months | HbA1c | I-7.89 ± 1.93  C-7.83 ± 1.91 | I-25.7(3.5)  C-25.6(3.6) | NR | I-8.71 (3.49)  C- 8.57 (3.38) | NR | I-2.84 ± 0.91)  C-2.83 ± 0.91 |
| 6 | Kleinman NJ 2017^23^ | India | I-44  C-46 | I-10.0(5–16)  C- 8.5(4–14) | I-48.8–9.0  C-48.0–9.5 | **Intervention** F- 18.2(8)  M- NR  **Control-**  F-41.3(19)  M- NR | Participants received the m Health app and a mobile phone data stipend | Usual hospital care | 6 months | HbA1c and Improved medication adherence and frequency of blood glucose self-testing. | I-9.4 ± 1.2  C-9.1–1.1 | I-29.7 ± 6.0  C-28.0 ± 4.2 | I-79.5 (35)  C-93.5 (43) | I- 185.1 ± 67.7  C- 166.5 ± 54.4 | NR | NR |
| 7 | Lazo-Porras 2020^24^ | Peru | I-86  C-86 | I-13.3 (8.5)  C-12.7 (7.9) | I- 60.3 (9.2)  C-62.1 (9.8) | **Intervention** F- 52 (60.5)  M- NR  **Control-**  F-56 (65.1)  M- NR | SMS and voice messages were delivered by the automated system | Attended only the initiation visit | 18  months | Additional mHealth-to-foot thermometry was not effective in reducing foot ulceration. HbA1c was assessed at baseline only | I-  C-8.2 (1.9)  C-8.9 (2.3) | I-28.0 (4.4) %  C-27.9 (4.8) % | NR | NR | NR | NR |
| 8 | Li J 2021^25^ | China | I- 55 (54.5)  C-46 (45.5) | I-44.7 (42.5)  C- 49.7 (40.6) | I-45.3 (11.0)  C- 51.7 (8.6) | **Intervention** F- 12 (22)  M- NR  **Control-**  F-12 (26)  M- NR | Asked to engage in moderate to vigorous physical activity for at least 150 minutes per week; each participant was prescribed individualized exercises.  In addition, a mHealth app and chest band was given | Asked to engage in moderate to vigorous physical activity for at least 150 minutes per week; each participant was prescribed individualized exercises. Did not receive an app or a chest band and self-reported exercise intensity and duration. | 3 months | Physical activity, HbA1c level, and increased cardiopulmonary endurance. | I-7.2 ± 1.8  C-7.5 ± 1.8 | I- 25.4 (4.0)  C- 25.0 (3.1) | NR | NR | NR | NR |
| 9 | Sanaeinasab H 2021^26^ | Iran | I-40  C-40 | **<5**  I-23 (57.5)  C- 26 (65.0)  ≥5  I-17 (42.5)  C-14 (35.0) | **<50**  I- 9 (22.5)  C-12 (30.0)  **≥50**  I- 31 (77.5)  C-28 (70.0) | **Intervention** F- 22 (55.0)  M- 18 (45.0)  **Control-**  F-25 (62.5)  M- 15 (37.5) | intervention consisted of six educational sessions on diabetes self-care held over 45 days with short videos. The Educational strategies included role playing, group discussion, watching a short video, and listening to a lecture on a weekly basis using small groups. | Received routine diabetic care at the clinic | 1.5 months | Lifestyle measures improved in the intervention group, whereas only the nutrition domain enhanced in the control group and HbA1c | I- 8.8 ± 1.6  C-8.9 ± 1.7 | I- 29.2 (2.8)  C-29.1 (2.7) | NR | I-214.0 (57.7)  C-218.6 (55.9) | I-51.3 (14.6)  C-48.2 (13.8) | I-135.4 (30.6)  C-137.5 (32.0) |
| 10 | Molavynejad S 2022^27^ | Mongolia | I-123  C-120 | I-6.34 ± 5.12  C- 5.43 ± 5.38 | I- 45.88 ± 9.09  C-48.31 ± 7.61 | **Intervention** F- 73(59.3%)  M-50(40.7%)  **Control-**  F-55(45.8%)  M-65(54.2%) | The educational movie was displayed in the  Clinic. At the end there was a discussion using  a question-and-answer format. The total movie  length was two hours.  DVDs or Cell phone formats were used for distance learning.  Also, reminders for the participants were sent via text messages to watch the video education again and to get back if they had any questions. | received oral face-to-face education via group sessions. Four educational sessions during a two-week interval via lectures | 12 months | Changes in patients’ weight, glycaemic parameters, Lipid profiles and  HbA1c | I-7.90±1.88  C-7.82±1.88 | I-93.76±15.28  C-92.52±16.17 | NR | I-166.65±83.54  C-162.32±79.57 | I-42.59±7.18  C-43.72±7.39 | I-108.59±26.14  C- 113.81±28.19 |
| 11 | Van Olmen J 2017^28^ | Africa, Cambodia & Philippines | I-401  C- 380 | I-4(2–8)  C-NR | I-58±10  C-NR | **Intervention** F- NR  M-29  **Control-**  F-NR  M-NR | Self-management education as well as a mobile phone for self-management support (DSME+DSMS) | Self-management education, was provided (DSME-only) | 24 months | HbA1c | I-8.2±2.1  C-NR | I-+0.0±1.9,  C-+0.0±2.4, | Change  I- -15.0%  C--10.4%, | NR | NR | NR |
| 12 | Wang X 2020^29^ | China | I-85  C-86 | NR | I-55.4(9.7)  C-54.7(11.8) | **Intervention** F- 43  M-42  **Control-**  F-31  M-55 | evaluated messages on diabetes self-care | Regular diabetes self-care-related education | 12 months | FPG and behavioural outcomes | I-9.2 ± 2.0  C-9.1 ± 2.0 | I-25.9(3.5)  C-25.8(4.1) | I-9.2(2.8)  C-8.8(2.4) | NR | I- 1.1(0.2)  C-1.1(0.3) | I-3.0(0.9)  C-2.9(1.0) |
| 13 | Wang Y 2019^30^ | China | I-60  C-60 | NR | I-45.13 ± 7.83  C-45.8 ± 8.38 | **Intervention** F- 27  M-33  **Control-**  F-29  M-31 | Continuous care based on the mobile phone application | Conventional care | 6 months | HbA1c | I-8.62 ± 2.33  C-8.68 ± 2.26 | NR | I-9.41 ± 4.32  C- 9.21 ± 4.16 | NR | NR | NR |
| 14 | Xu Z et al 2020^31^ | China | I-36  C-40 | NR | I-46.0 (44.0-48.2)  C-47.5 (44.0-55.0) | **Intervention** F- 18 (50.0）  M-18 (50.0）  **Control-**  F-16 (40.0）  M-24 (60.0） | Educational content via DHealth Bar. Push notifications twice per week on WeChat and had access to the educational content | Educational content via printed handbook | 6 months | Energy, fat, and carbohydrate intake, physical activity | NR | I-25.3 (24.7-26.2)  C-24.7 (23.4-26.1) | NR | NR | NR | NR |
| 15 | Yu Y 2019^32^ | China | I-93  C-92 | NR | I-50.3–10.4  C-51.4–9.4 | **Intervention** F- 14  M-31  **Control-**  F-24  M-21 | mobile phone application (MPA) combined with self-monitoring of blood glucose (SMBG) | Without app and only SMBG | 6 months | HbA1c | I-8.7 ± 1.9 | I-26.0–3.9  C-26.1–4.6 | I-7.9–2.2  C-8.1–2.6 | NR | I-1.1–0.3  C-1.3–0.5 | I-2.6–1.1  C-3.3–1.1 |
| 16 | Zhou W 2016^33^ | China | I-50  C-50 | I-6.65±5.14  C-6.63±5.06 | I-55.0±13.1  C-53.5±12.4 | **Intervention** F- 23  M-27  **Control-**  F-20  M-30 | The Welltang intervention group received training for the use of Welltang | usual standard of care | 3months | HbA1c diabetes knowledge, and self-care behaviours. | I-9.86 ±2.38  C-9.76±2.51 | I-23.04±4.09  C-23.01±4.04 | NR | NR | NR | NR |
| 17 | Zhang Y 2018^34^ | China | 998 | I-10.5(10.0)  C-11.1(8.8) | I-50.8(14.3)  C-52.6(13.2) | **Intervention** F- 50.7(43.2–58.2)  M-NR  **Control-**  F-48.9(40.3–57.5)  M-NR | Systematic health education model | Conventional model groups | 24months | HbA1c, LDL cholesterol and BMI | I- 7.86 ± 1.2  C-8.15 ± 1.5 | I-25.3(4.5)  C-26.6(6.2) | NR | NR | I-62.3(15.9)  C-61.2(19.5) | I-128.8(9.6)  C-131.2(9.5) |
| 18 | Zhai 2020^35^ | China | I-60  C-58 | 11.7 | I-54.12±11.1  C-55.64±14.2 | **Intervention** F- NR  M-30 (52.6%)  **Control-**  F-NR  M-28 (51.8%) | mHealth app and conventional diabetic treatment | conventional diabetic treatment | 6months | HbA1c, behavioural | I-8.86±1.15  C-9.05 ± 1.23 | I-26.41±3.41  C-25.57±3.33 | I-8.16±2.57  C-8.54±2.91 | I-12.11±3.01  C-11.09±2.32 | NR | I-3.25±1.06  C-3.41±0.74 |
| 19 | Guo, M. 2023 ^36^ | China | I-32  C-32 | NA | I– 55.25 ± 13.80  C-59.46 ± 14.59 | **Intervention** F- 9 (28.13)  M-23 (71.88)  **Control-**  F-16 (50.00)  M-16 (50.00) | implantable glucose sensor and a mobile app featuring guidance from general practitioners | The control group received usual health management | 1 month | HbA1c, BMI and FBG | I-8.97 ± 2.12  C-9.14 ± 1.96 | I-24.00 ± 3.13  C-24.32 ± 3.14 | NA | I-7.81 ± 1.77  C-8.11 ± 1.41 | NA | NA |
| 20 | Zhang, P.  2024 ^37^ | China | I-40 facilities  C-40 | I- 9.1 (7.04)  C-9.0(6.68) | I-61.4 (7.1)  C-61.6(6.9) | **Intervention** F- NR  M-449 (43.3%)  **Control-**  F-NR  M-481 (46.5%) | digital health application | Control arm clusters received usual care without access to the digital health application | 24 months | HbA1c, BMI, FBG and LDL | I-8.59 (1.40)  C-8.66 (1.48) | I- 26.6 (3.6)  C-26.7(3.3) | NA | I-9.83 (3.06)  C- 9.74 (3.14) | NA | I- 3.33 (0.87)  C- 3.29 (0.84) |
| 21 | Eghtedari, M. 2023 ^38^ | Iran | I-32  C-32 | I-10.83(7.34)  C-11.12(7.21) | I-55.93(10.97)  C-61.09(9.37) | **Intervention** F and  M-1.60(0.50)  **Control-**  F and M-1.53(0.51) | web-based program that included multimedia educational content (such as videos, lectures, educational motion graphics, text files, posters, and podcasts) | The control group received usual care | 21 days | HbA1c | I- [-0.99(1.01)]  C- [-0.26(1.43)] | NA | I-6.20(0.41)  C-5.96(0.41) | NA | NA | NA |
| 22 | Anjali, M. 2023 ^39^ | India | I-53  C-53 | I-24(45.3)  C-26(49.1) | I-55.30(10.5)  C-51.45(9.9) | **Intervention** F- 14(26.4)  M-39(73.6)  **Control-**  F-19(35.8)  M- 34(64.2) | The intervention group received DSME in addition to routine OPD care | The control group received only routine outpatient department (OPD) care | 3 months | HbA1c, BMI and FBG | I-9.0(2.2)  C-9.9(3.1) | I-25.2(4.6)  C-27.7(6.3) | I-48(1.1)  C-4.9(1.2) | I-171.8(66.1)  C-191.9(97.2) | NA | NA |
| 23 | Tang H 2025 ^40^ | China | I-68  C-68 | I-26 (37.1)  C-23 (33.8) | I-50 (71.4)  C-46 (67.7) | **Intervention** F- 18 (25.7) M-52 (74.3)  **Control-**  F-26 (38.2)  M-42 (61.8) | mHealth app and uric acid tester, a smart blood glucose meter, along with weekly phone reminders | usual standard of care with a blood glucose meter and a logbook | 3 and 6 months | HbA1c, BP, BMI, SMBG, and satisfaction with the APP. | I-7.9 (7.1, 9.5)  C-8.4 (7.3, 10.0) | I-25.2 (3.9)  C-24.9 (4.4) | NA | NA | NA | NA |
