# Supplementary material for: Effect of digital health interventions on glycaemic control among type 2 diabetes individuals in low and middle-income countries: a systematic review and meta-analysis
Source: BMC Public Health. 2026 Jun 8;26:2340. doi: 10.1186/s12889-026-27908-x (PMC13463556; doi:10.1186/s12889-026-27908-x)
Supplement: Supplementary file 2 [file 12889_2026_27908_MOESM2_ESM.docx]

**Supplementary File 4**

Table 1: Sensitivity analysis for the outcomes assessed in the study

| **Outcome** | **Studies (n)** | **Effect estimate MD [95%CI]** | **P value** | **Sensitivity analysis** | **Tau^2^** | **Cochrane test (p value)** | **I ^2^ (%)** | **Egger’s test (p)** |
| --- | --- | --- | --- | --- | --- | --- | --- | --- |
| HbA1c | 15 | -0.58 [-0.83, -0.33] | <0.00001 | -0.58 [-0.83; -0.34] | 0.18 | <0.00001 | 98 | 0.511 |
| FPG | 12 | -0.50 [-0.84, -0.17] | 0.003 | -0.52 [-1.23; 0.18] | 0.15 | <0.00001 | 78 | 0.988 |
| BMI | 9 | -0.09 [-0.37, 0.20] | 0.56 | -0.09 [-0.39; 0.20] | 0.06 | 0.10 | 40 | 0.036 |
| Total cholesterol | 3 | -1.03 [-11.58, 9.53] | 0.85 | -0.92 [-11.18; 9.35] | 55.29 | 0.04 | 68 | 0.238 |
| HDL | 4 | 0.97 [-4.07, 6.00] | 0.71 | 0.93 [-3.90; 5.75] | 19.28 | 0.002 | 79 | 0.217 |
| LDL | 7 | -2.10 [-2.90, -1.30] | <0.00001 | -3.13 [-6.76; 0.50] | 0.65 | <0.00001 | 99 | 0.267 |
| Triglycerides | 3 | -18.15 [-34.18, -2.11] | 0.03 | -18.14[-34.18; -2.11] | 0.00 | 0.49 | 0 | 0.192 |
| BGM | 4 | -0.25 [-1.12, 0.62] | 0.58 | -0.24 [-1.08; 0.60] | 0.47 | 0.001 | 82 | 0.473 |
| Physical activity | 5 | 0.33 [-0.81, 1.46] | 0.57 | 0.33 [-0.77; 1.43] | 1.63 | <0.00001 | 100 | 0.306 |
| Medication adherence | 4 | -0.38 [-1.18, 0.41] | 0.34 | -0.39 [-1.1; 0.31] | 0.40 | 0.0005 | 83 | 0.618 |

Table 2: Reporting Certainty of Evidence among the included studies

| **Outcome** | **Studies (n)/ Participants** | **Effect Estimate MD (95% CI)** | **Risk of Bias** | **Inconsistency** | **Indirectness** | **Imprecision** | **Publication Bias** | **Certainty of Evidence** |
| --- | --- | --- | --- | --- | --- | --- | --- | --- |
| HbA1c | 15/ 23,941 | -0.58 [-0.83, -0.33] | Serious | Not serious | Serious | Not serious | Not serious | ⨁⨁◯◯ Low |
| FPG | 12/ 22,874 | -0.50 [-0.84, -0.17] | Serious | Not serious | Serious | Not serious | Not serious | ⨁⨁◯◯ Low |
| BMI | 9/ 2506 | -0.09 [-0.37, 0.20] | Serious | Not serious | Not serious | Very serious | Serious | ⨁◯◯◯ Very Low |
| Total cholesterol | 3/ 1297 | -1.03 [-11.58, 9.53] | Not serious | Not serious | Serious | Very serious | Not serious | ⨁◯◯◯ Very Low |
| HDL | 4/ 1402 | 0.97 [-4.07, 6.00] | Not serious | Not serious | Serious | Very serious | Not serious | ⨁◯◯◯ Very Low |
| LDL | 7/ 23120 | -2.10 [-2.90, -1.30] | Serious | Not serious | Not serious | Serious | Not serious | ⨁⨁◯◯ Low |
| Triglycerides | 3/ 424 | -18.15 [-34.18, -2.11] | Serious | Not serious | Not serious | Serious | Not serious | ⨁⨁◯◯ Low |
| BGM | 4/ 467 | -0.25 [-1.12, 0.62] | Serious | Not serious | Not serious | Serious | Not serious | ⨁⨁◯◯ Low |
| Physical activity | 5/ 437 | 0.33 [-0.81, 1.46] | Serious | Not serious | Serious | Very serious | Not serious | ⨁◯◯◯ Very Low |
| Medication Adherence | 4/345 | -0.38 [-1.18, 0.41] | Serious | Not serious | Serious | Very serious | Not serious | ⨁◯◯◯ Very Low |
