# Supplementary material for: Effect of digital health interventions on glycaemic control among type 2 diabetes individuals in low and middle-income countries: a systematic review and meta-analysis
Source: BMC Public Health. 2026 Jun 8;26:2340. doi: 10.1186/s12889-026-27908-x (PMC13463556; doi:10.1186/s12889-026-27908-x)
Supplement: Supplementary file 3 [file 12889_2026_27908_MOESM3_ESM.docx]

**Search log**

| PubMed | ((((adult onset diabetes mellitus[MeSH Terms]) OR (type 2 diabetes mellitus[MeSH Terms])) OR ("non‐insulin dependent diabetes mellitus"[Title/Abstract] OR "type 2 diabetes mellitus"[Title/Abstract] OR "adult onset diabetes mellitus"[Title/Abstract])) AND ((("mobile applications"[MeSH Terms]) OR ("mobile applications"[Title/Abstract] OR "mhealth app"[Title/Abstract] OR "phone application"[Title/Abstract])) OR ("digital health intervention"[Title/Abstract]))) AND (("afghanistan"[MeSH Terms] OR "afghanistan"[All Fields] OR "afghanistan s"[All Fields] OR ("albania"[MeSH Terms] OR "albania"[All Fields]) OR ("algeria"[MeSH Terms] OR "algeria"[All Fields]) OR "american samoa"[All Fields] OR ("angola"[MeSH Terms] OR "angola"[All Fields] OR "angola s"[All Fields]) OR ("argentina"[MeSH Terms] OR "argentina"[All Fields] OR "argentina s"[All Fields] OR "argentinae"[All Fields]) OR ("argentina"[MeSH Terms] OR "argentina"[All Fields] OR "argentina s"[All Fields] OR "argentinae"[All Fields]) OR ("armenia"[MeSH Terms] OR "armenia"[All Fields]) OR ("azerbaijan"[MeSH Terms] OR "azerbaijan"[All Fields]) OR ("bangladesh"[MeSH Terms] OR "bangladesh"[All Fields] OR "bangladesh s"[All Fields]) OR ("republic of belarus"[MeSH Terms] OR ("republic"[All Fields] AND "belarus"[All Fields]) OR "republic of belarus"[All Fields] OR "belarus"[All Fields]) OR ("belize"[MeSH Terms] OR "belize"[All Fields]) OR ("benin"[MeSH Terms] OR "benin"[All Fields] OR "benin s"[All Fields]) OR ("bhutan"[MeSH Terms] OR "bhutan"[All Fields] OR "bhutan s"[All Fields]) OR ("bolivia"[MeSH Terms] OR "bolivia"[All Fields]) OR (("BOSNIA AND HERZEGOVINA"[MeSH Terms] OR ("bosnia"[All Fields] AND "herzegovina"[All Fields]) OR "BOSNIA AND HERZEGOVINA"[All Fields] OR "bosnia"[All Fields]) AND "BOSNIA AND HERZEGOVINA"[MeSH Terms]) OR "BOSNIA AND HERZEGOVINA"[Title/Abstract] OR "botswana"[MeSH Terms] OR "botswana"[Title/Abstract] OR "brazil"[MeSH Terms] OR "brazil"[Title/Abstract] OR "bulgaria"[MeSH Terms] OR "bulgaria"[Title/Abstract] OR "burkina faso"[MeSH Terms] OR "burkina faso"[Title/Abstract] OR "burundi"[MeSH Terms] OR "burundi"[Title/Abstract] OR "cabo verde"[MeSH Terms] OR "cabo verde"[Title/Abstract] OR "cambodia"[MeSH Terms] OR "cambodia"[Title/Abstract] OR "cameroon"[MeSH Terms] OR "cameroon"[Title/Abstract] OR "central african republic"[MeSH Terms] OR "central african republic"[Title/Abstract] OR "chad"[MeSH Terms] OR "chad"[Title/Abstract] OR "china"[MeSH Terms] OR "china"[Title/Abstract] OR "colombia"[MeSH Terms] OR "colombia"[Title/Abstract] OR "comoros"[MeSH Terms] OR "comoros"[Title/Abstract] OR (("congo"[MeSH Terms] OR "congo"[All Fields]) AND "dem"[All Fields] AND "rep"[Title/Abstract]) OR "costa rica"[MeSH Terms] OR "costa rica"[Title/Abstract] OR "cote d ivoire"[MeSH Terms] OR "cote d ivoire"[Title/Abstract] OR "cuba"[MeSH Terms] OR "cuba"[Title/Abstract] OR "djibouti"[MeSH Terms] OR "djibouti"[Title/Abstract] OR "dominica"[MeSH Terms] OR "dominica"[Title/Abstract] OR "dominican republic"[MeSH Terms] OR "dominican republic"[Title/Abstract] OR "ecuador"[MeSH Terms] OR "ecuador"[Title/Abstract] OR "el salvador"[MeSH Terms] OR "el salvador"[Title/Abstract] OR "equatorial guinea"[MeSH Terms] OR "equatorial guinea"[Title/Abstract] OR "egypt"[MeSH Terms] OR "arab republic of egypt"[Title/Abstract] OR "eritrea"[MeSH Terms] OR "eritrea"[Title/Abstract] OR "ethiopia"[MeSH Terms] OR "ethiopia"[Title] OR "fiji"[MeSH Terms] OR "fiji"[Title/Abstract] OR "gabon"[MeSH Terms] OR "gabon"[Title/Abstract] OR "gambia"[MeSH Terms] OR "gambia"[Title/Abstract] OR ("georgia"[MeSH Terms] OR "georgia republic"[MeSH Terms]) OR "georgia"[Title/Abstract] OR "ghana"[MeSH Terms] OR "ghana"[Title/Abstract] OR "grenada"[MeSH Terms] OR "grenada"[Title/Abstract] OR "guatemala"[MeSH Terms] OR "guatemala"[Title/Abstract] OR "guinea bissau"[MeSH Terms] OR "guinea bissau"[Title/Abstract] OR "guyana"[MeSH Terms] OR "guyana"[Title] OR "haiti"[MeSH Terms] OR "haiti"[Title] OR "honduras"[MeSH Terms] OR "honduras"[Title/Abstract] OR "India"[MeSH Terms] OR "India"[Title/Abstract] OR "indonesia"[MeSH Terms] OR "indonesia"[Title/Abstract] OR "iran"[MeSH Terms] OR "iran"[Title/Abstract] OR "jamaica"[MeSH Terms] OR "jamaica"[Title/Abstract] OR "jordan"[MeSH Terms] OR "jordan"[Title/Abstract] OR "jordan"[MeSH Terms] OR "jordan"[Title] OR "kazakhstan"[MeSH Terms] OR "kazakhstan"[Title/Abstract] OR "kenya"[MeSH Terms] OR "kenya"[Title] OR "kiribati"[MeSH Terms] OR "kiribati"[Title] OR "democratic people s republic of korea"[MeSH Terms] OR "democratic people s republic of korea"[Title/Abstract] OR "kosovo"[MeSH Terms] OR "kosovo"[Title/Abstract] OR "kyrgyzstan"[MeSH Terms] OR "kyrgyz republic"[Title/Abstract] OR "lao pdr"[Title/Abstract] OR "lebanon"[MeSH Terms] OR "lebanon"[Title/Abstract] OR "lesotho"[MeSH Terms] OR "lesotho"[Title/Abstract] OR "liberia"[MeSH Terms] OR "liberia"[Title] OR "libya"[MeSH Terms] OR "libya"[Title/Abstract] OR "madagascar"[MeSH Terms] OR "madagascar"[Title/Abstract] OR "malawi"[MeSH Terms] OR "malawi"[Title] OR "malaysia"[MeSH Terms] OR "malaysia"[Title/Abstract] OR "maldives"[MeSH Terms] OR "maldives"[Title] OR "Mali"[MeSH Terms] OR "Mali"[Title/Abstract] OR "micronesia"[MeSH Terms] OR "marshall islands"[Title/Abstract] OR "mauritania"[MeSH Terms] OR "mauritania"[Title/Abstract] OR "mauritius"[MeSH Terms] OR "mauritius"[Title] OR "mexico"[MeSH Terms] OR "mexico"[Title/Abstract] OR "micronesia"[MeSH Terms] OR "micronesia"[Title/Abstract] OR "moldova"[MeSH Terms] OR "moldova"[Title/Abstract] OR "mongolia"[MeSH Terms] OR "mongolia"[Title/Abstract] OR "montenegro"[MeSH Terms] OR "montenegro"[Title/Abstract] OR "morocco"[MeSH Terms] OR "morocco"[Title/Abstract] OR "mozambique"[MeSH Terms] OR "mozambique"[Title/Abstract] OR "myanmar"[MeSH Terms] OR "myanmar"[Title/Abstract] OR "namibia"[MeSH Terms] OR "namibia"[Title/Abstract] OR "nepal"[MeSH Terms] OR "nepal"[Title/Abstract] OR "nicaragua"[MeSH Terms] OR "nicaragua"[Title/Abstract] OR "niger"[MeSH Terms] OR "niger"[Title/Abstract] OR "nigeria"[MeSH Terms] OR "nigeria"[Title/Abstract] OR "republic of north macedonia"[MeSH Terms] OR "macedonia"[Title/Abstract] OR "pakistan"[MeSH Terms] OR "pakistan"[Title/Abstract] OR "panama"[MeSH Terms] OR "panama"[Title/Abstract] OR "papua new guinea"[MeSH Terms] OR "papua new guinea"[Title/Abstract] OR "paraguay"[MeSH Terms] OR "paraguay"[Title/Abstract] OR "peru"[MeSH Terms] OR "peru"[Title/Abstract] OR "philippines"[MeSH Terms] OR "philippines"[Title/Abstract] OR "romania"[MeSH Terms] OR "romania"[Title/Abstract] OR "russia"[MeSH Terms] OR "russian federation"[Title/Abstract] OR "rwanda"[MeSH Terms] OR "rwanda"[Title/Abstract] OR "samoa"[MeSH Terms] OR "samoa"[Title/Abstract] OR "sao tome and principe"[MeSH Terms] OR ("sao tome"[Title/Abstract] AND "principe"[Title/Abstract]) OR "senegal"[MeSH Terms] OR "senegal"[Title/Abstract] OR "serbia"[MeSH Terms] OR "serbia"[Title/Abstract] OR "sierra leone"[MeSH Terms] OR "sierra leone"[Title/Abstract] OR "melanesia"[MeSH Terms] OR "solomon islands"[Title/Abstract] OR "somalia"[MeSH Terms] OR "somalia"[Title/Abstract] OR "south africa"[MeSH Terms] OR "south africa"[Title/Abstract] OR "Sudan"[MeSH Terms] OR "Sudan"[Title/Abstract] OR "sri lanka"[MeSH Terms] OR "sri lanka"[Title/Abstract] OR "saint lucia"[MeSH Terms] OR "st lucia"[Title/Abstract] OR "saint vincent and the grenadines"[MeSH Terms] OR ("st vincent"[Title/Abstract] AND "the grenadines"[Title/Abstract]) OR "south sudan"[MeSH Terms] OR "south sudan"[Title/Abstract] OR "suriname"[MeSH Terms] OR "suriname"[Title/Abstract] OR (("syrian people"[Supplementary Concept] OR "syrian people"[All Fields] OR "syrian"[All Fields] OR "mesocricetus"[MeSH Terms] OR "mesocricetus"[All Fields] OR "syrians"[All Fields]) AND "arb"[All Fields] AND "republic"[Title/Abstract]) OR "tajikistan"[MeSH Terms] OR "tajikistan"[Title/Abstract] OR "tanzania"[MeSH Terms] OR "tanzania"[Title/Abstract] OR "thailand"[MeSH Terms] OR "thailand"[Title/Abstract] OR "timor leste"[MeSH Terms] OR "timor leste"[Title/Abstract] OR "togo"[MeSH Terms] OR "togo"[Title/Abstract] OR "Tonga"[MeSH Terms] OR "Tonga"[Title/Abstract] OR "Tunisia"[MeSH Terms] OR "Tunisia"[Title/Abstract] OR "turkey"[MeSH Terms] OR "turkey"[Title/Abstract] OR "turkmenistan"[MeSH Terms] OR "turkmenistan"[Title/Abstract] OR "micronesia"[MeSH Terms] OR "tuvalu"[Title/Abstract] OR "uganda"[MeSH Terms] OR "uganda"[Title/Abstract] OR "ukraine"[MeSH Terms] OR "ukraine"[Title/Abstract] OR "uzbekistan"[MeSH Terms] OR "uzbekistan"[Title/Abstract] OR "vanuatu"[MeSH Terms] OR "vanuatu"[Title/Abstract] OR "vietnam"[MeSH Terms] OR "vietnam"[Title/Abstract] OR ("west bank"[Title/Abstract] AND "gaza"[Title/Abstract]) OR "yemen"[MeSH Terms] OR "yemen"[Title/Abstract] OR "zambia"[MeSH Terms] OR "zambia"[MeSH Terms] OR "zimbabwe"[MeSH Terms] OR "zimbabwe"[Title/Abstract])) AND (1000/1/1:2025/5/1[pdat]) |
| --- | --- |
| CENTRAL (Cochrane Central Register of Controlled Trials) | (((([mh "adult onset diabetes mellitus"]) OR ([mh "type 2 diabetes mellitus"])) OR ("non‐insulin dependent diabetes mellitus":ti,ab OR "type 2 diabetes mellitus":ti,ab OR "adult onset diabetes mellitus":ti,ab)) AND ((([mh "mobile applications"]) OR ("mobile applications":ti,ab OR "mhealth app":ti,ab OR "phone application":ti,ab)) OR ("digital health intervention":ti,ab))) AND (([mh afghanistan] OR afghanistan OR "afghanistan s" OR ([mh albania] OR albania) OR ([mh algeria] OR algeria) OR "american samoa" OR ([mh angola] OR angola OR "angola s") OR ([mh argentina] OR argentina OR "argentina s" OR argentinae) OR ([mh argentina] OR argentina OR "argentina s" OR argentinae) OR ([mh armenia] OR armenia) OR ([mh azerbaijan] OR azerbaijan) OR ([mh bangladesh] OR bangladesh OR "bangladesh s") OR ([mh "republic of belarus"] OR (republic AND belarus) OR "republic of belarus" OR belarus) OR ([mh belize] OR belize) OR ([mh benin] OR benin OR "benin s") OR ([mh bhutan] OR bhutan OR "bhutan s") OR ([mh bolivia] OR bolivia) OR (([mh "BOSNIA AND HERZEGOVINA"] OR (bosnia AND herzegovina) OR "BOSNIA AND HERZEGOVINA" OR bosnia) AND [mh "BOSNIA AND HERZEGOVINA"]) OR "BOSNIA AND HERZEGOVINA":ti,ab OR [mh botswana] OR botswana:ti,ab OR [mh brazil] OR brazil:ti,ab OR [mh bulgaria] OR bulgaria:ti,ab OR [mh "burkina faso"] OR "burkina faso":ti,ab OR [mh burundi] OR burundi:ti,ab OR [mh "cabo verde"] OR "cabo verde":ti,ab OR [mh cambodia] OR cambodia:ti,ab OR [mh cameroon] OR cameroon:ti,ab OR [mh "central african republic"] OR "central african republic":ti,ab OR [mh chad] OR chad:ti,ab OR [mh china] OR china:ti,ab OR [mh colombia] OR colombia:ti,ab OR [mh comoros] OR comoros:ti,ab OR (([mh congo] OR congo) AND dem AND rep:ti,ab) OR [mh "costa rica"] OR "costa rica":ti,ab OR [mh "cote d ivoire"] OR "cote d ivoire":ti,ab OR [mh cuba] OR cuba:ti,ab OR [mh djibouti] OR djibouti:ti,ab OR [mh dominica] OR dominica:ti,ab OR [mh "dominican republic"] OR "dominican republic":ti,ab OR [mh ecuador] OR ecuador:ti,ab OR [mh "el salvador"] OR "el salvador":ti,ab OR [mh "equatorial guinea"] OR "equatorial guinea":ti,ab OR [mh egypt] OR "arab republic of egypt":ti,ab OR [mh eritrea] OR eritrea:ti,ab OR [mh ethiopia] OR ethiopia:ti OR [mh fiji] OR fiji:ti,ab OR [mh gabon] OR gabon:ti,ab OR [mh gambia] OR gambia:ti,ab OR ([mh georgia] OR [mh "georgia republic"]) OR georgia:ti,ab OR [mh ghana] OR ghana:ti,ab OR [mh grenada] OR grenada:ti,ab OR [mh guatemala] OR guatemala:ti,ab OR [mh "guinea bissau"] OR "guinea bissau":ti,ab OR [mh guyana] OR guyana:ti OR [mh haiti] OR haiti:ti OR [mh honduras] OR honduras:ti,ab OR [mh India] OR India:ti,ab OR [mh indonesia] OR indonesia:ti,ab OR [mh iran] OR iran:ti,ab OR [mh jamaica] OR jamaica:ti,ab OR [mh jordan] OR jordan:ti,ab OR [mh jordan] OR jordan:ti OR [mh kazakhstan] OR kazakhstan:ti,ab OR [mh kenya] OR kenya:ti OR [mh kiribati] OR kiribati:ti OR [mh "democratic people s republic of korea"] OR "democratic people s republic of korea":ti,ab OR [mh kosovo] OR kosovo:ti,ab OR [mh kyrgyzstan] OR "kyrgyz republic":ti,ab OR "lao pdr":ti,ab OR [mh lebanon] OR lebanon:ti,ab OR [mh lesotho] OR lesotho:ti,ab OR [mh liberia] OR liberia:ti OR [mh libya] OR libya:ti,ab OR [mh madagascar] OR madagascar:ti,ab OR [mh malawi] OR malawi:ti OR [mh malaysia] OR malaysia:ti,ab OR [mh maldives] OR maldives:ti OR [mh Mali] OR Mali:ti,ab OR [mh micronesia] OR "marshall islands":ti,ab OR [mh mauritania] OR mauritania:ti,ab OR [mh mauritius] OR mauritius:ti OR [mh mexico] OR mexico:ti,ab OR [mh micronesia] OR micronesia:ti,ab OR [mh moldova] OR moldova:ti,ab OR [mh mongolia] OR mongolia:ti,ab OR [mh montenegro] OR montenegro:ti,ab OR [mh morocco] OR morocco:ti,ab OR [mh mozambique] OR mozambique:ti,ab OR [mh myanmar] OR myanmar:ti,ab OR [mh namibia] OR namibia:ti,ab OR [mh nepal] OR nepal:ti,ab OR [mh nicaragua] OR nicaragua:ti,ab OR [mh niger] OR niger:ti,ab OR [mh nigeria] OR nigeria:ti,ab OR [mh "republic of north macedonia"] OR macedonia:ti,ab OR [mh pakistan] OR pakistan:ti,ab OR [mh panama] OR panama:ti,ab OR [mh "papua new guinea"] OR "papua new guinea":ti,ab OR [mh paraguay] OR paraguay:ti,ab OR [mh peru] OR peru:ti,ab OR [mh philippines] OR philippines:ti,ab OR [mh romania] OR romania:ti,ab OR [mh russia] OR "russian federation":ti,ab OR [mh rwanda] OR rwanda:ti,ab OR [mh samoa] OR samoa:ti,ab OR [mh "sao tome and principe"] OR ("sao tome":ti,ab AND principe:ti,ab) OR [mh senegal] OR senegal:ti,ab OR [mh serbia] OR serbia:ti,ab OR [mh "sierra leone"] OR "sierra leone":ti,ab OR [mh melanesia] OR "solomon islands":ti,ab OR [mh somalia] OR somalia:ti,ab OR [mh "south africa"] OR "south africa":ti,ab OR [mh Sudan] OR Sudan:ti,ab OR [mh "sri lanka"] OR "sri lanka":ti,ab OR [mh "saint lucia"] OR "st lucia":ti,ab OR [mh "saint vincent and the grenadines"] OR ("st vincent":ti,ab AND "the grenadines":ti,ab) OR [mh "south sudan"] OR "south sudan":ti,ab OR [mh suriname] OR suriname:ti,ab OR (("syrian people":kw OR "syrian people" OR syrian OR [mh mesocricetus] OR mesocricetus OR syrians) AND arb AND republic:ti,ab) OR [mh tajikistan] OR tajikistan:ti,ab OR [mh tanzania] OR tanzania:ti,ab OR [mh thailand] OR thailand:ti,ab OR [mh "timor leste"] OR "timor leste":ti,ab OR [mh togo] OR togo:ti,ab OR [mh Tonga] OR Tonga:ti,ab OR [mh Tunisia] OR Tunisia:ti,ab OR [mh turkey] OR turkey:ti,ab OR [mh turkmenistan] OR turkmenistan:ti,ab OR [mh micronesia] OR tuvalu:ti,ab OR [mh uganda] OR uganda:ti,ab OR [mh ukraine] OR ukraine:ti,ab OR [mh uzbekistan] OR uzbekistan:ti,ab OR [mh vanuatu] OR vanuatu:ti,ab OR [mh vietnam] OR vietnam:ti,ab OR ("west bank":ti,ab AND gaza:ti,ab) OR [mh yemen] OR yemen:ti,ab OR [mh zambia] OR [mh zambia] OR [mh zimbabwe] OR zimbabwe:ti,ab)) |
| EMBASE (via ELSEVIER) | (((('adult onset diabetes mellitus'/exp) OR ('type 2 diabetes mellitus'/exp)) OR ('non‐insulin dependent diabetes mellitus':ti,ab OR 'type 2 diabetes mellitus':ti,ab OR 'adult onset diabetes mellitus':ti,ab)) AND ((('mobile applications'/exp) OR ('mobile applications':ti,ab OR 'mhealth app':ti,ab OR 'phone application':ti,ab)) OR ('digital health intervention':ti,ab))) AND ((afghanistan/exp OR afghanistan OR 'afghanistan s' OR (albania/exp OR albania) OR (algeria/exp OR algeria) OR 'american samoa' OR (angola/exp OR angola OR 'angola s') OR (argentina/exp OR argentina OR 'argentina s' OR argentinae) OR (argentina/exp OR argentina OR 'argentina s' OR argentinae) OR (armenia/exp OR armenia) OR (azerbaijan/exp OR azerbaijan) OR (bangladesh/exp OR bangladesh OR 'bangladesh s') OR ('republic of belarus'/exp OR (republic AND belarus) OR 'republic of belarus' OR belarus) OR (belize/exp OR belize) OR (benin/exp OR benin OR 'benin s') OR (bhutan/exp OR bhutan OR 'bhutan s') OR (bolivia/exp OR bolivia) OR (('BOSNIA AND HERZEGOVINA'/exp OR (bosnia AND herzegovina) OR 'BOSNIA AND HERZEGOVINA' OR bosnia) AND 'BOSNIA AND HERZEGOVINA'/exp) OR 'BOSNIA AND HERZEGOVINA':ti,ab OR botswana/exp OR botswana:ti,ab OR brazil/exp OR brazil:ti,ab OR bulgaria/exp OR bulgaria:ti,ab OR 'burkina faso'/exp OR 'burkina faso':ti,ab OR burundi/exp OR burundi:ti,ab OR 'cabo verde'/exp OR 'cabo verde':ti,ab OR cambodia/exp OR cambodia:ti,ab OR cameroon/exp OR cameroon:ti,ab OR 'central african republic'/exp OR 'central african republic':ti,ab OR chad/exp OR chad:ti,ab OR china/exp OR china:ti,ab OR colombia/exp OR colombia:ti,ab OR comoros/exp OR comoros:ti,ab OR ((congo/exp OR congo) AND dem AND rep:ti,ab) OR 'costa rica'/exp OR 'costa rica':ti,ab OR 'cote d ivoire'/exp OR 'cote d ivoire':ti,ab OR cuba/exp OR cuba:ti,ab OR djibouti/exp OR djibouti:ti,ab OR dominica/exp OR dominica:ti,ab OR 'dominican republic'/exp OR 'dominican republic':ti,ab OR ecuador/exp OR ecuador:ti,ab OR 'el salvador'/exp OR 'el salvador':ti,ab OR 'equatorial guinea'/exp OR 'equatorial guinea':ti,ab OR egypt/exp OR 'arab republic of egypt':ti,ab OR eritrea/exp OR eritrea:ti,ab OR ethiopia/exp OR ethiopia:ti OR fiji/exp OR fiji:ti,ab OR gabon/exp OR gabon:ti,ab OR gambia/exp OR gambia:ti,ab OR (georgia/exp OR 'georgia republic'/exp) OR georgia:ti,ab OR ghana/exp OR ghana:ti,ab OR grenada/exp OR grenada:ti,ab OR guatemala/exp OR guatemala:ti,ab OR 'guinea bissau'/exp OR 'guinea bissau':ti,ab OR guyana/exp OR guyana:ti OR haiti/exp OR haiti:ti OR honduras/exp OR honduras:ti,ab OR India/exp OR India:ti,ab OR indonesia/exp OR indonesia:ti,ab OR iran/exp OR iran:ti,ab OR jamaica/exp OR jamaica:ti,ab OR jordan/exp OR jordan:ti,ab OR jordan/exp OR jordan:ti OR kazakhstan/exp OR kazakhstan:ti,ab OR kenya/exp OR kenya:ti OR kiribati/exp OR kiribati:ti OR 'democratic people s republic of korea'/exp OR 'democratic people s republic of korea':ti,ab OR kosovo/exp OR kosovo:ti,ab OR kyrgyzstan/exp OR 'kyrgyz republic':ti,ab OR 'lao pdr':ti,ab OR lebanon/exp OR lebanon:ti,ab OR lesotho/exp OR lesotho:ti,ab OR liberia/exp OR liberia:ti OR libya/exp OR libya:ti,ab OR madagascar/exp OR madagascar:ti,ab OR malawi/exp OR malawi:ti OR malaysia/exp OR malaysia:ti,ab OR maldives/exp OR maldives:ti OR Mali/exp OR Mali:ti,ab OR micronesia/exp OR 'marshall islands':ti,ab OR mauritania/exp OR mauritania:ti,ab OR mauritius/exp OR mauritius:ti OR mexico/exp OR mexico:ti,ab OR micronesia/exp OR micronesia:ti,ab OR moldova/exp OR moldova:ti,ab OR mongolia/exp OR mongolia:ti,ab OR montenegro/exp OR montenegro:ti,ab OR morocco/exp OR morocco:ti,ab OR mozambique/exp OR mozambique:ti,ab OR myanmar/exp OR myanmar:ti,ab OR namibia/exp OR namibia:ti,ab OR nepal/exp OR nepal:ti,ab OR nicaragua/exp OR nicaragua:ti,ab OR niger/exp OR niger:ti,ab OR nigeria/exp OR nigeria:ti,ab OR 'republic of north macedonia'/exp OR macedonia:ti,ab OR pakistan/exp OR pakistan:ti,ab OR panama/exp OR panama:ti,ab OR 'papua new guinea'/exp OR 'papua new guinea':ti,ab OR paraguay/exp OR paraguay:ti,ab OR peru/exp OR peru:ti,ab OR philippines/exp OR philippines:ti,ab OR romania/exp OR romania:ti,ab OR russia/exp OR 'russian federation':ti,ab OR rwanda/exp OR rwanda:ti,ab OR samoa/exp OR samoa:ti,ab OR 'sao tome and principe'/exp OR ('sao tome':ti,ab AND principe:ti,ab) OR senegal/exp OR senegal:ti,ab OR serbia/exp OR serbia:ti,ab OR 'sierra leone'/exp OR 'sierra leone':ti,ab OR melanesia/exp OR 'solomon islands':ti,ab OR somalia/exp OR somalia:ti,ab OR 'south africa'/exp OR 'south africa':ti,ab OR Sudan/exp OR Sudan:ti,ab OR 'sri lanka'/exp OR 'sri lanka':ti,ab OR 'saint lucia'/exp OR 'st lucia':ti,ab OR 'saint vincent and the grenadines'/exp OR ('st vincent':ti,ab AND 'the grenadines':ti,ab) OR 'south sudan'/exp OR 'south sudan':ti,ab OR suriname/exp OR suriname:ti,ab OR (('syrian people':tn OR 'syrian people' OR syrian OR mesocricetus/exp OR mesocricetus OR syrians) AND arb AND republic:ti,ab) OR tajikistan/exp OR tajikistan:ti,ab OR tanzania/exp OR tanzania:ti,ab OR thailand/exp OR thailand:ti,ab OR 'timor leste'/exp OR 'timor leste':ti,ab OR togo/exp OR togo:ti,ab OR Tonga/exp OR Tonga:ti,ab OR Tunisia/exp OR Tunisia:ti,ab OR turkey/exp OR turkey:ti,ab OR turkmenistan/exp OR turkmenistan:ti,ab OR micronesia/exp OR tuvalu:ti,ab OR uganda/exp OR uganda:ti,ab OR ukraine/exp OR ukraine:ti,ab OR uzbekistan/exp OR uzbekistan:ti,ab OR vanuatu/exp OR vanuatu:ti,ab OR vietnam/exp OR vietnam:ti,ab OR ('west bank':ti,ab AND gaza:ti,ab) OR yemen/exp OR yemen:ti,ab OR zambia/exp OR zambia/exp OR zimbabwe/exp OR zimbabwe:ti,ab)) |
| CINAHL | AB (diabetes mellitus or type 2 diabetes mellitus) AND TI (self management education or education or diabetes self management education program ) OR TI ( mhealth or mobile health or m-health or mobile app or mobile application or smartphone application or app or apps ) AND TI ( low and middle income countries )  • English Language  • Abstract Available  • Human  • Clinical Trial  • Journal Article  • Research  • Review |
| Scopus | ((((INDEXTERMS("adult onset diabetes mellitus")) OR (INDEXTERMS("type 2 diabetes mellitus"))) OR (TITLE-ABS("non‐insulin dependent diabetes mellitus") OR TITLE-ABS("type 2 diabetes mellitus") OR TITLE-ABS("adult onset diabetes mellitus"))) AND (((INDEXTERMS("mobile applications")) OR (TITLE-ABS("mobile applications") OR TITLE-ABS("mhealth app") OR TITLE-ABS("phone application"))) OR (TITLE-ABS("digital health intervention")))) AND ((INDEXTERMS(afghanistan) OR ALL(afghanistan) OR ALL("afghanistan s") OR (INDEXTERMS(albania) OR ALL(albania)) OR (INDEXTERMS(algeria) OR ALL(algeria)) OR ALL("american samoa") OR (INDEXTERMS(angola) OR ALL(angola) OR ALL("angola s")) OR (INDEXTERMS(argentina) OR ALL(argentina) OR ALL("argentina s") OR ALL(argentinae)) OR (INDEXTERMS(argentina) OR ALL(argentina) OR ALL("argentina s") OR ALL(argentinae)) OR (INDEXTERMS(armenia) OR ALL(armenia)) OR (INDEXTERMS(azerbaijan) OR ALL(azerbaijan)) OR (INDEXTERMS(bangladesh) OR ALL(bangladesh) OR ALL("bangladesh s")) OR (INDEXTERMS("republic of belarus") OR (ALL(republic) AND ALL(belarus)) OR ALL("republic of belarus") OR ALL(belarus)) OR (INDEXTERMS(belize) OR ALL(belize)) OR (INDEXTERMS(benin) OR ALL(benin) OR ALL("benin s")) OR (INDEXTERMS(bhutan) OR ALL(bhutan) OR ALL("bhutan s")) OR (INDEXTERMS(bolivia) OR ALL(bolivia)) OR ((INDEXTERMS("BOSNIA AND HERZEGOVINA") OR (ALL(bosnia) AND ALL(herzegovina)) OR ALL("BOSNIA AND HERZEGOVINA") OR ALL(bosnia)) AND INDEXTERMS("BOSNIA AND HERZEGOVINA")) OR TITLE-ABS("BOSNIA AND HERZEGOVINA") OR INDEXTERMS(botswana) OR TITLE-ABS(botswana) OR INDEXTERMS(brazil) OR TITLE-ABS(brazil) OR INDEXTERMS(bulgaria) OR TITLE-ABS(bulgaria) OR INDEXTERMS("burkina faso") OR TITLE-ABS("burkina faso") OR INDEXTERMS(burundi) OR TITLE-ABS(burundi) OR INDEXTERMS("cabo verde") OR TITLE-ABS("cabo verde") OR INDEXTERMS(cambodia) OR TITLE-ABS(cambodia) OR INDEXTERMS(cameroon) OR TITLE-ABS(cameroon) OR INDEXTERMS("central african republic") OR TITLE-ABS("central african republic") OR INDEXTERMS(chad) OR TITLE-ABS(chad) OR INDEXTERMS(china) OR TITLE-ABS(china) OR INDEXTERMS(colombia) OR TITLE-ABS(colombia) OR INDEXTERMS(comoros) OR TITLE-ABS(comoros) OR ((INDEXTERMS(congo) OR ALL(congo)) AND ALL(dem) AND TITLE-ABS(rep)) OR INDEXTERMS("costa rica") OR TITLE-ABS("costa rica") OR INDEXTERMS("cote d ivoire") OR TITLE-ABS("cote d ivoire") OR INDEXTERMS(cuba) OR TITLE-ABS(cuba) OR INDEXTERMS(djibouti) OR TITLE-ABS(djibouti) OR INDEXTERMS(dominica) OR TITLE-ABS(dominica) OR INDEXTERMS("dominican republic") OR TITLE-ABS("dominican republic") OR INDEXTERMS(ecuador) OR TITLE-ABS(ecuador) OR INDEXTERMS("el salvador") OR TITLE-ABS("el salvador") OR INDEXTERMS("equatorial guinea") OR TITLE-ABS("equatorial guinea") OR INDEXTERMS(egypt) OR TITLE-ABS("arab republic of egypt") OR INDEXTERMS(eritrea) OR TITLE-ABS(eritrea) OR INDEXTERMS(ethiopia) OR TITLE(ethiopia) OR INDEXTERMS(fiji) OR TITLE-ABS(fiji) OR INDEXTERMS(gabon) OR TITLE-ABS(gabon) OR INDEXTERMS(gambia) OR TITLE-ABS(gambia) OR (INDEXTERMS(georgia) OR INDEXTERMS("georgia republic")) OR TITLE-ABS(georgia) OR INDEXTERMS(ghana) OR TITLE-ABS(ghana) OR INDEXTERMS(grenada) OR TITLE-ABS(grenada) OR INDEXTERMS(guatemala) OR TITLE-ABS(guatemala) OR INDEXTERMS("guinea bissau") OR TITLE-ABS("guinea bissau") OR INDEXTERMS(guyana) OR TITLE(guyana) OR INDEXTERMS(haiti) OR TITLE(haiti) OR INDEXTERMS(honduras) OR TITLE-ABS(honduras) OR INDEXTERMS(India) OR TITLE-ABS(India) OR INDEXTERMS(indonesia) OR TITLE-ABS(indonesia) OR INDEXTERMS(iran) OR TITLE-ABS(iran) OR INDEXTERMS(jamaica) OR TITLE-ABS(jamaica) OR INDEXTERMS(jordan) OR TITLE-ABS(jordan) OR INDEXTERMS(jordan) OR TITLE(jordan) OR INDEXTERMS(kazakhstan) OR TITLE-ABS(kazakhstan) OR INDEXTERMS(kenya) OR TITLE(kenya) OR INDEXTERMS(kiribati) OR TITLE(kiribati) OR INDEXTERMS("democratic people s republic of korea") OR TITLE-ABS("democratic people s republic of korea") OR INDEXTERMS(kosovo) OR TITLE-ABS(kosovo) OR INDEXTERMS(kyrgyzstan) OR TITLE-ABS("kyrgyz republic") OR TITLE-ABS("lao pdr") OR INDEXTERMS(lebanon) OR TITLE-ABS(lebanon) OR INDEXTERMS(lesotho) OR TITLE-ABS(lesotho) OR INDEXTERMS(liberia) OR TITLE(liberia) OR INDEXTERMS(libya) OR TITLE-ABS(libya) OR INDEXTERMS(madagascar) OR TITLE-ABS(madagascar) OR INDEXTERMS(malawi) OR TITLE(malawi) OR INDEXTERMS(malaysia) OR TITLE-ABS(malaysia) OR INDEXTERMS(maldives) OR TITLE(maldives) OR INDEXTERMS(Mali) OR TITLE-ABS(Mali) OR INDEXTERMS(micronesia) OR TITLE-ABS("marshall islands") OR INDEXTERMS(mauritania) OR TITLE-ABS(mauritania) OR INDEXTERMS(mauritius) OR TITLE(mauritius) OR INDEXTERMS(mexico) OR TITLE-ABS(mexico) OR INDEXTERMS(micronesia) OR TITLE-ABS(micronesia) OR INDEXTERMS(moldova) OR TITLE-ABS(moldova) OR INDEXTERMS(mongolia) OR TITLE-ABS(mongolia) OR INDEXTERMS(montenegro) OR TITLE-ABS(montenegro) OR INDEXTERMS(morocco) OR TITLE-ABS(morocco) OR INDEXTERMS(mozambique) OR TITLE-ABS(mozambique) OR INDEXTERMS(myanmar) OR TITLE-ABS(myanmar) OR INDEXTERMS(namibia) OR TITLE-ABS(namibia) OR INDEXTERMS(nepal) OR TITLE-ABS(nepal) OR INDEXTERMS(nicaragua) OR TITLE-ABS(nicaragua) OR INDEXTERMS(niger) OR TITLE-ABS(niger) OR INDEXTERMS(nigeria) OR TITLE-ABS(nigeria) OR INDEXTERMS("republic of north macedonia") OR TITLE-ABS(macedonia) OR INDEXTERMS(pakistan) OR TITLE-ABS(pakistan) OR INDEXTERMS(panama) OR TITLE-ABS(panama) OR INDEXTERMS("papua new guinea") OR TITLE-ABS("papua new guinea") OR INDEXTERMS(paraguay) OR TITLE-ABS(paraguay) OR INDEXTERMS(peru) OR TITLE-ABS(peru) OR INDEXTERMS(philippines) OR TITLE-ABS(philippines) OR INDEXTERMS(romania) OR TITLE-ABS(romania) OR INDEXTERMS(russia) OR TITLE-ABS("russian federation") OR INDEXTERMS(rwanda) OR TITLE-ABS(rwanda) OR INDEXTERMS(samoa) OR TITLE-ABS(samoa) OR INDEXTERMS("sao tome and principe") OR (TITLE-ABS("sao tome") AND TITLE-ABS(principe)) OR INDEXTERMS(senegal) OR TITLE-ABS(senegal) OR INDEXTERMS(serbia) OR TITLE-ABS(serbia) OR INDEXTERMS("sierra leone") OR TITLE-ABS("sierra leone") OR INDEXTERMS(melanesia) OR TITLE-ABS("solomon islands") OR INDEXTERMS(somalia) OR TITLE-ABS(somalia) OR INDEXTERMS("south africa") OR TITLE-ABS("south africa") OR INDEXTERMS(Sudan) OR TITLE-ABS(Sudan) OR INDEXTERMS("sri lanka") OR TITLE-ABS("sri lanka") OR INDEXTERMS("saint lucia") OR TITLE-ABS("st lucia") OR INDEXTERMS("saint vincent and the grenadines") OR (TITLE-ABS("st vincent") AND TITLE-ABS("the grenadines")) OR INDEXTERMS("south sudan") OR TITLE-ABS("south sudan") OR INDEXTERMS(suriname) OR TITLE-ABS(suriname) OR ((CHEM(term) OR ALL("syrian people") OR ALL(syrian) OR INDEXTERMS(mesocricetus) OR ALL(mesocricetus) OR ALL(syrians)) AND ALL(arb) AND TITLE-ABS(republic)) OR INDEXTERMS(tajikistan) OR TITLE-ABS(tajikistan) OR INDEXTERMS(tanzania) OR TITLE-ABS(tanzania) OR INDEXTERMS(thailand) OR TITLE-ABS(thailand) OR INDEXTERMS("timor leste") OR TITLE-ABS("timor leste") OR INDEXTERMS(togo) OR TITLE-ABS(togo) OR INDEXTERMS(Tonga) OR TITLE-ABS(Tonga) OR INDEXTERMS(Tunisia) OR TITLE-ABS(Tunisia) OR INDEXTERMS(turkey) OR TITLE-ABS(turkey) OR INDEXTERMS(turkmenistan) OR TITLE-ABS(turkmenistan) OR INDEXTERMS(micronesia) OR TITLE-ABS(tuvalu) OR INDEXTERMS(uganda) OR TITLE-ABS(uganda) OR INDEXTERMS(ukraine) OR TITLE-ABS(ukraine) OR INDEXTERMS(uzbekistan) OR TITLE-ABS(uzbekistan) OR INDEXTERMS(vanuatu) OR TITLE-ABS(vanuatu) OR INDEXTERMS(vietnam) OR TITLE-ABS(vietnam) OR (TITLE-ABS("west bank") AND TITLE-ABS(gaza)) OR INDEXTERMS(yemen) OR TITLE-ABS(yemen) OR INDEXTERMS(zambia) OR INDEXTERMS(zambia) OR INDEXTERMS(zimbabwe) OR TITLE-ABS(zimbabwe))) |
| Web of Science | TI=("type 2 diabetes mellitus" AND "self-management" AND "Mobile Application*" OR "m-health" AND "Low and Middle Income Countr*") |
