# Supplementary material for: Effect of digital health interventions on glycaemic control among type 2 diabetes individuals in low and middle-income countries: a systematic review and meta-analysis
Source: BMC Public Health. 2026 Jun 8;26:2340. doi: 10.1186/s12889-026-27908-x (PMC13463556; doi:10.1186/s12889-026-27908-x)
Supplement: Supplementary file 4 [file 12889_2026_27908_MOESM4_ESM.docx]

**Forest plots and funnel plots**

**Figure 4:** Forest plot showing the change in Fasting Blood Glucose (FBG) levels in the Digital Health Intervention group compared to the control group

**Figure 5:** Forest plot showing the change in Body Mass Index (BMI) levels in the Digital Health Intervention group compared to the control group

**Figure 6:** Forest plot showing the change in HDL levels in the Digital Health Intervention group compared to the control group

**Figure 7:** Forest plot showing the change in LDL levels in the Digital Health Intervention group compared to the control group

**Figure 8:** Forest plot showing the change in Triglyceride (TG) levels in the Digital Health Intervention group compared to the control group

**Figure 9:** Forest plot showing the change in Total Cholesterol (TC) levels in the Digital Health Intervention group compared to the control group

**Figure 10:** Forest plot showing the change in Blood Glucose Monitoring (BGM) in the Digital Health Intervention group compared to the control group

**Figure 11:** Forest plot showing the change in Physical Activity in the Digital Health Intervention group compared to the control group

**Figure 12:** Forest plot showing the change in Medication Adherence in the Digital Health Intervention group compared to the control group

**Funnel plots**

**Figure S1: Funnel plot showing the distribution of the studies on HbA1c as the outcome**

**Figure S2: Funnel plot showing the distribution of the studies on FBG as the outcome**

**Figure S3: Funnel plot showing the distribution of the studies on BMI as the outcome**

**Figure S4: Funnel plot showing the distribution of the studies on HDL as the outcome**

**Figure S5: Funnel plot showing the distribution of the studies on LDL as the outcome**

**Figure S6: Funnel plot showing the distribution of the studies on TC as the outcome**

**Figure S7: Funnel plot showing the distribution of the studies on TG as the outcome**

**Figure S8: Funnel plot showing the distribution of the studies on BGM as the outcome**

**Figure S9: Funnel plot showing the distribution of the studies on Physical activity as the outcome**

**Figure S10: Funnel plot showing the distribution of the studies on Medication Adherence as the outcome**
